# Py-CoMFA, docking, and molecular dynamics simulations of *Leishmania (L.) amazonensis* arginase inhibitors

**DOI:** 10.1038/s41598-024-62520-2

**Published:** 2024-05-21

**Authors:** Priscila Goes Camargo, Carine Ribeiro dos Santos, Magaly Girão Albuquerque, Carlos Rangel Rodrigues, Camilo Henrique da Silva Lima

**Affiliations:** 1 Faculdade de Farmácia, Departamento de Fármacos e Medicamentos, Universidade Federal do Rio de Janeiro, Rio de Janeiro, RJ Brazil; 2 Laboratório de Modelagem Molecular (LabMMol), Instituto de Química, Universidade Federal do Rio de Janeiro, Rio de Janeiro, RJ Brazil

**Keywords:** 3D-QSAR, Molecular interaction fields, Molecular docking, Molecular dynamics, Antileishmanial, Neglected diseases, Medicinal chemistry, Chemistry, Chemical biology, Cheminformatics, Organic chemistry, Infectious diseases

## Abstract

Leishmaniasis is a disease caused by a protozoan of the genus *Leishmania*, affecting millions of people, mainly in tropical countries, due to poor social conditions and low economic development. First-line chemotherapeutic agents involve highly toxic pentavalent antimonials, while treatment failure is mainly due to the emergence of drug-resistant strains. *Leishmania* arginase (ARG) enzyme is vital in pathogenicity and contributes to a higher infection rate, thus representing a potential drug target. This study helps in designing ARG inhibitors for the treatment of leishmaniasis. Py-CoMFA (3D-QSAR) models were constructed using 34 inhibitors from different chemical classes against ARG from *L. (L.) amazonensis* (*La*ARG). The 3D-QSAR predictions showed an excellent correlation between experimental and calculated pIC_50_ values. The molecular docking study identified the favorable hydrophobicity contribution of phenyl and cyclohexyl groups as substituents in the enzyme allosteric site. Molecular dynamics simulations of selected protein–ligand complexes were conducted to understand derivatives’ interaction modes and affinity in both active and allosteric sites. Two cinnamide compounds, **7g** and **7k**, were identified, with similar structures to the reference **4h** allosteric site inhibitor. These compounds can guide the development of more effective arginase inhibitors as potential antileishmanial drugs.

## Introduction

Human leishmaniasis are a group of vector-borne diseases, transmitted by the bite of infected female phlebotomine sandflies, caused by protozoan parasites comprising more than 20 species of the genus *Leishmania*^1^. According to the clinical manifestations, there are three main forms of leishmaniasis (ICD-11 code: 1F54): (i) visceral leishmaniasis (VL), which is fatal if left untreated in over 95% of cases; (ii) cutaneous leishmaniasis (CL), which is the most common form; and (iii) mucosal/mucocutaneous leishmaniasis (ML or MCL)^2^. Globally, leishmaniasis is among the top 10 neglected tropical diseases (NTDs), it is endemic in 99 tropical and subtropical countries, with more than 12 million people infected^3^. Over 90% of new cases have been reported in Brazil, Ethiopia, Somalia, Sudan, and India in the past 4 years^4^. This disease is primarily associated with poor social conditions and low economic development since poverty dramatically impacts human health^5^.

To date, there is no licensed vaccine against human leishmaniasis^6^. The treatment against all *Leishmania* forms still involves using chemotherapeutic agents, mainly administered parenterally (intravenous, intramuscular or intralesional), including pentavalent antimony (Sb^V^) compounds as first-line drugs, such as meglumine antimoniate and sodium stibogluconate, which are highly toxic, and non-antimony-based compounds as second-line drugs, such as amphotericin B and pentamidine, which also have severe side effects^7^. Miltefosine, another example of a non-antimony-based compound, despite being the only drug for oral use against leishmaniasis, presents teratogenic risk^8–10^. The failures of current drug therapy are partially due to the emergence of drug resistant *Leishmania* strains^11^. Therefore, it is crucial to discover new antileishmanial chemotherapeutic agents, more effective, and selective. According to the Drugs for Neglected Diseases initiative (DNDi)^12^, the target product profile (TPP) for new antileishmanial chemotherapeutic agents includes activity against all species of *Leishmania*, efficacy in treatment of less than two weeks, preferably a once-daily oral drug, and safer than available treatment^9^.

In the pursuit of discovering novel drug candidates, molecular targets that are crucial for the survival of parasites have been thoroughly investigated to identify compounds that are both selective and less toxic^13^. One noteworthy target from this research is the arginase (ARG) enzyme, also known as L-arginine aminohydrolase (EC 3.5.3.1), which has shown promise as a potential target in developing new antitrypanosomal drugs^4,14–16^.

ARG is a manganese-dependent metalloenzyme that catalyzes the hydrolyzes of L-arginine into L-ornithine and urea, while the L-ornithine product is a precursor of polyamines necessary to produce trypanothione, a vital antioxidant agent that controls reactive oxygen species (ROS)^17–21^. This makes L-ornithine a crucial component of the redox defense mechanism of parasites. The expression and activity of ARG in *Leishmania* species contribute to a higher infection rate and play a vital role in the pathogenicity of the parasite^4,14^. An increase in ARG activity reduces L-arginine availability to the nitric oxide synthase (NOS) enzyme^4^. This, in turn, diminishes the formation of nitric oxide (NO), which lowers the host’s defensive capacity and increases the parasite’s infectivity. Simply put, the ARG enzyme can negatively regulate the levels of NO produced in the body by consuming the NOS enzyme substrate. It is worth noting that scientific studies have shown that ARG inhibitors can reduce the ability of *Leishmania* spp. to establish an infection in macrophages^14^.

Computer-aided drug design (CADD) techniques, including ligand-based drug design (LBDD) and structure-based drug design (SBDD) approaches, are widely applied in the drug discovery process^22^. The field of quantitative structure–activity relationships (QSAR) modeling, whether through regression or classification methods, aims to correlate chemical structures with their biological responses, and it is mostly applied as an LBDD approach^23,24^. However, the traditional approach of using topological (1D and/or 2D) molecular descriptors alone does not consider the tridimensional (3D) geometric features of molecules, which can lead to difficulties adequately describing interactions between ligands and receptors^25,26^. Therefore, a tridimensional QSAR (3D-QSAR) method can be applied to overcome these limitations for better results.

Comparative Molecular Field Analysis (CoMFA)^27^ is a 3D-QSAR ligand-based and alignment-dependent method developed by Cramer et al.^27^ that utilizes statistical techniques to correlate molecular (steric and electrostatic) interaction fields (MIFs), which are interaction energies calculated with virtual probes in a 3D space, such as in the GRID method developed by Goodford^28^ with biological response endpoints ^29^. Ragno et al. (2020) developed a Python implementation of the CoMFA method, named Py-CoMFA, which is publicly available through the www.3d-qsar.com web portal to build 3D-QSAR models^30,31^. The resulting accuracy data can help understand the (quantitative) structure–activity relationships (Q)SAR that are most relevant for developing antileishmanial agents^32,33^.

On the other hand, a SBDD approach, such as the ligand-receptor molecular docking^34^ and molecular dynamics (MD) simulations methods^35^, combined with a LBDD approach, such as the CoMFA method (3D-QSAR)^36,37^, have paved the way for significant advancements in drug design. These computational tools have enabled researchers to study bioactive substances’ molecular mechanisms of action and have become a crucial strategy for identifying new hits^38^. In addition, molecular docking is necessary to understand the structural requirements and to analyze the interactions between ligands and receptors^39^, and in addition, to derive ligand-receptor complexes for further studies, such as molecular dynamics (MD) simulations, which may provide information about the stability of promising compounds analyzing the ligand-receptor complexes in the aqueous system^40^.

Furthermore, it is worth highlighting that this study aims to apply the Py-CoMFA (3D-QSAR) modeling method to a set of 34 compounds recently reported in the literature from 2018 to 2023 as inhibitors of the *Leishmania* (*L.*) *amazonensis* arginase (*La*ARG) enzyme, a relevant target of the parasite life cycle. During the cited period, there was a shortage of literature publications that incorporated a complete in silico study with multiple techniques such as 3D-QSAR (especially Py-CoMFA), molecular docking, and MD simulations for investigating arginase inhibitors, principally those focused targeting arginase from *L. (L.) amazonensis*, which has a greater ability to resist macrophages, neutrophils, and leishmanicidal drugs than other species of *Leishmania*^41,42^. After that, structure–activity relationship studies helped us to analyze the influence of aliphatic groups, with or without heteroatoms, and the presence of the cyclohexyl, pyrrole, and indole rings as side chains. Finally, the putative binding modes of the most active inhibitors were investigated by molecular docking on the *La*ARG enzyme, while the most relevant protein–ligand complexes were submitted to molecular dynamics simulations to verify the dynamic behavior of the putative interaction modes and binding affinities of those derivatives.

## Results and discussion

### Py-CoMFA (3D-QSAR) modeling

**Figure 1:**
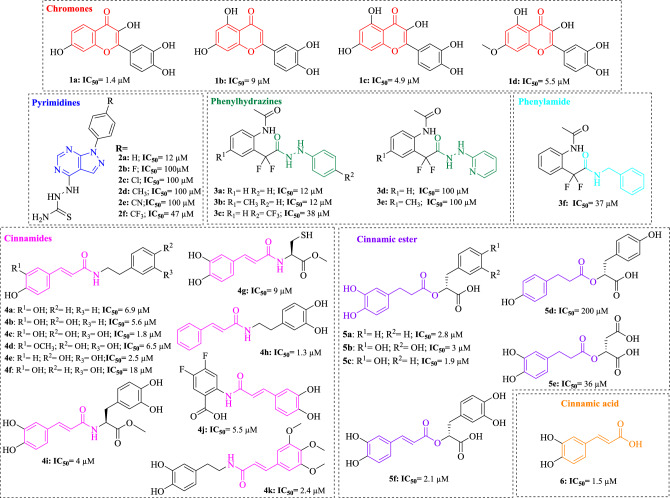
Chemical structures and half-maximal inhibitory concentration (IC_50_, μM) values of the *L. (L.) amazonensis* arginase inhibitors used in the Py-CoMFA (3D-QSAR) study. The classes of compounds are highlighted by different colors.

Py-CoMFA (3D-QSAR) models were constructed with 34 inhibitors reported in the literature between 2018 and 2023 against arginase from *L. (L) amazonensis* (*La*ARG). These 34 inhibitors belong to the chemical classes of chromones (**1a–1d**)^43^, pyrazolo-pyrimidines (**2a**–**2f**)^44^, phenylhydrazines or phenylamide (**3a**–**3f**)^45^, cinnamides (cinnamic acid amides or cinnamides) (**4a**–**4k**)^4^, and esters (**5a–5f**)^46^ derived from 3,4-dihydroxycinnamic acid (caffeic acid)^47^ (**6**) (Fig. 1).

Considering a traditional qualitative SAR analysis, it has been observed that cinnamic acid (**6**) and its derivatives, cinnamides (**4a–k**) and cinnamic esters (**5a–f**), are the most promising compounds that act as arginase inhibitors. Their IC_50_ values range from 1.3 to 200 µM. Among these compounds, cinnamic acid itself (**6**) and disubstituted cinnamide derivatives with acetylated groups (**4i, 4g**) or hydroxy or dihydroxybenzene (**4a–4f**) have been shown to have the strongest inhibitory activity (Fig. 1). Another group of inhibitors that exhibit exceptional inhibitory activity are chromones (**1a–1d**). These are also substituted with dihydroxybenzene with an IC_50_ range of 1.4 to 9 µM. It is evident that among these compounds, β-carbonyl hydroxylation significantly reduces the inhibitory activity of chromones **1b–d** by up to six times. The non-β-carbonyl hydroxylated derivative **1a**, on the other hand, exhibits the highest inhibitory potency (IC_50_ = 1.4 µM) (Fig. 1). Nitrogen derivatives like pyrimidines (**2a–f**), phenylhydrazines (**3a–e**), and phenylamide (**3f**) exhibit moderate inhibitory activity with IC_50_ values ranging from 12 to 100 µM. However, the biological activity appears to improve when the phenylhydrazine nucleus is included in the structural framework, with values ranging from 12 to 38 µM (Fig. 1).

It is important to keep in mind that Py-CoMFA models, as with other 3D-QSAR methods, can be affected by various factors that can impact their accuracy. Some of these factors include the conformational analysis method, putative bioactive conformation selection, and alignment rule, which is unfavorable to noncovalent molecular interactions^48^. Therefore, ensuring that the molecular target being evaluated in the model is carefully studied is crucial the probe atom variations need to be assessed^49^, and the set of molecules should be well-defined to obtain reliable results. It is important to note that hydrophobicity is not well quantified^50^, and thus, structures with high hydrophobicity can lead to misinterpretations. Additionally, the use of CoMFA is appropriate only with in vitro data.

**Figure 2:**
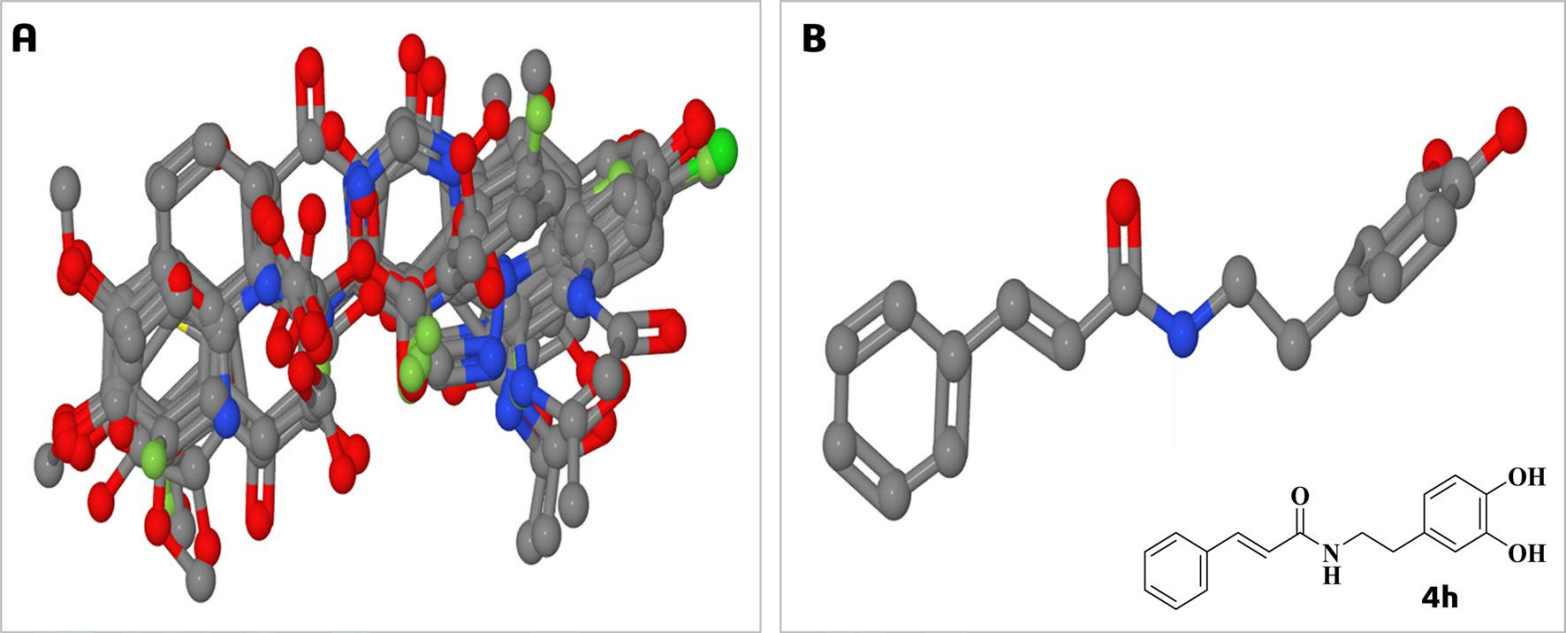
(**A**) Superimposed structures of all inhibitors under study. Compounds are shown in ball-and-stick model, and the atoms are color coded as follows carbon (grey), oxygen (red), nitrogen (blue), fluorine (green), and sulfur (yellow), while hydrogen atoms were omitted for clarity. (**B**) Longest conformation of **4 h** used as alignment template. Figure generated with Py-Align and Py-ConfSearch, respectively.

In this way, we first performed in the 3D-QSAR server (https://www.3d-qsar.com/), a conformational analysis of all inhibitors using the RDkit method with the UFF force field through the Py-ConfSearch module. The most extended conformation of each inhibitor was selected as the putative bioactive conformation, since in several crystal structures of *L. mexicana* arginase (LmARG), a series of boronic amino-acid inhibitors (PDB ID: 5HJ9, 5HJA, 4IU0, 4IU4) adopts an extended conformation. The same occurs for a substrate analog amino-acid inhibitor (PDB ID: 4IU1) and the L-ornithine product (PDB ID: 4IU5). Then, we aligned the inhibitors (Fig. 2A) using the Py-Align module, applying the RDkit method using the most extended conformation of the most active inhibitor **4h** (IC_50_ = 1.3 µM) as an alignment template (Fig. 2B).

Once the dataset compounds are aligned, we utilize two methods to split the dataset into training and test sets. While most QSAR studies, including CoMFA, generally apply random splitting, we also wanted to investigate the potential differences when implementing a supervised approach, such as the Kennard-Stone method ^51^. Twenty-five compounds were used as the training set for deriving the QSAR models, and nine compounds were used as the test set for external validation (Tables 1, 2).

**Table 1:** Statistical parameters of the Py-CoMFA (3D-QSAR) models built using steric field (STE) and 2 Å grid spacing, including Y-randomization (Y-r) test, according to the training and test sets splitting method (random or Kennard-Stone) and the probe atoms and charges (C*sp*^3^, + 1; O*sp*^3^, − 1; and H, + 1).

Models	Random	Kennard-Stone	Kennard-Stone	Kennard-Stone
Probe atom	C*sp*^3^	C*sp*^3^	O*sp*^3^	H
Charge	+ 1	+ 1	− 1	+ 1
r^2^	0.995	0.999	0.992	0.953
SDEC	0.046	0.022	0.061	0.147
q^2^	0.469	0.573	0.513	0.499
SDEP	0.487	0.444	0.474	0.481
F-test	155.7	197.8	172.6	79.24
*r*	0.910	0.923	0.918	0.844
r^2^ (Y-r)	0.992	0.986	0.972	0.890
SDEC (Y-r)	0.056	0.082	0.114	0.225
q^2^ (Y-r)	0.121	0.103	− 0.198	− 0.201
SDEP (Y-r)	0.627	0.643	0.743	0.744

All models presented a *P* value < 0.0001. r^2^ = coefficient of determination; SDEC = standard deviation error in calculation; q^2^ = r^2^ from leave-one-out (LOO) cross-validation; SDEP = standard deviation error in prediction; *r* = Pearson correlation coefficient.

**Table 2:** Experimental pIC_50_ (pIC_50Exp_), predicted pIC_50_ (pIC_50Pred_), and residual (Res = pIC_50Exp_ − pIC_50Pred_) values for the training and test sets compounds based on the Py-CoMFA models built using steric field, 2 Å grid spacing, and the Kennard-Stone training and test sets splitting method, as probe atoms C*sp*^3^ (charge =  + 1), H (charge =  + 1), and O*sp*^3^ (charge =  − 1).

Classes	pIC_50Exp_	C*sp*^3^ (+ 1)		O*sp*^3^ (− 1)		H (+ 1)	
		pIC_50Pred_	Res	pIC_50Pred_	Res	pIC_50Pred_	Res
Chromones							
**1a**	5.854	5.858	− 0.004	5.837	0.017	5.911	− 0.057
*1b	5.046	5.757	− 0.712	5.716	− 0.670	5.805	− 0.759
**1c**	5.310	5.308	0.002	5.369	− 0.059	5.269	0.041
**1d**	5.260	5.265	− 0.006	5.233	0.027	5.254	0.006
Pyrimidines							
**2a**	4.921	4.918	0.003	4.918	0.003	4.611	0.310
**2b**	4.000	4.005	− 0.005	4.042	− 0.042	3.992	0.008
**2c**	4.000	4.019	− 0.019	3.973	0.027	4.054	− 0.054
**2d**	4.000	3.980	0.020	3.984	0.016	4.128	− 0.128
*2e	4.000	4.338	− 0.338	4.382	− 0.382	4.462	− 0.462
*2f	4.328	4.786	− 0.458	4.755	− 0.427	5.059	− 0.731
Phenylhydrazines							
**3a**	4.921	4.925	− 0.004	4.874	0.047	4.903	0.018
**3b**	4.921	4.936	− 0.015	4.924	− 0.003	4.800	0.121
**3c**	4.420	4.429	− 0.009	4.436	− 0.016	4.542	− 0.122
**3d**	4.000	3.994	0.006	3.990	0.010	4.165	− 0.165
**3e**	4.000	3.990	0.010	4.001	− 0.001	4.120	− 0.120
**3f**	4.432	4.433	− 0.009	4.415	0.017	4.310	0.122
Cinnamides							
**4a**	5.161	5.148	0.014	5.148	0.013	5.158	0.003
**4b**	5.252	5.266	− 0.014	5.107	0.145	5.156	0.096
*4c	5.745	5.726	0.019	5.808	− 0.063	5.615	0.130
*4d	5.187	5.639	− 0.452	5.579	− 0.392	5.527	− 0.340
**4e**	5.602	5.668	− 0.066	5.734	− 0.132	5.587	0.015
*4f	4.745	4.741	0.003	4.920	− 0.175	5.109	− 0.364
*4g	5.046	4.974	0.072	5.055	− 0.009	5.060	− 0.014
**4h**	5.886	5.811	0.075	5.791	0.095	5.543	0.343
*4i	5.398	4.841	0.557	4.585	0.813	4.508	0.890
**4j**	5.260	5.269	− 0.010	5.248	0.012	5.320	− 0.060
**4k**	5.620	5.624	− 0.004	5.627	− 0.007	5.493	0.127
Cinnamic esters							
**5a**	5.553	5.565	− 0.012	5.567	− 0.014	5.670	− 0.117
**5b**	5.523	5.503	0.020	5.481	0.042	5.664	− 0.141
**5c**	5.721	5.730	− 0.009	5.682	0.039	5.769	− 0.048
**5d**	3.699	3.696	0.003	3.720	− 0.021	3.524	0.175
**5e**	4.444	4.439	0.004	4.432	0.012	4.502	− 0.058
*5f	5.678	4.822	0.855	4.860	0.818	4.396	1.282
Cinnamic acid							
**6**	5.824	5.808	0.016	5.877	− 0.053	5.773	0.051

Res = Residuals *Test set compounds.

Compared to the random training and test sets splitting method, we found that the Kennard-Stone^51^ splitting method provided the compounds that show high dissimilarity in relation to the independent variables (descriptors) that were separated as a test set and the best parameters for our models. We explored three different fields, namely steric (STE), electrostatic (ELE), and the combination of both (STE + ELE) (see Table S1). However, we found that the models based on steric fields (STE) provided the best results (as shown in Table 1), and hence, we conducted all further analyses based on these models.

We also tested the models with different probe atoms and charges (C*sp*^3^, + 1; O*sp*^3^, − 1; and H, + 1) and found that all models performed well, showing statistical parameters indicating that the coefficient of determination (r^2^) was more significant than 0.9 for all models. In contrast, the r^2^ from the leave-one-out cross-validation (q^2^) was greater than 0.5 for models from the Kennard-Stone splitting method, considering C*sp*^3^ (+ 1) and O*sp*^3^ (− 1) as probe atoms, in addition to a Pearson correlation coefficient with positive value (*r* ≅ 1), i.e., variables are directly correlated^52^. However, the best model was for C*sp*^3^ (+ 1) as a probe atom, with higher r^2^ = 0.999 and q^2^ = 0.573 (Table 1). We also performed a Y-randomization test, which showed low values of q^2^, indicating that our developed 3D-QSAR models with original data are robust and not inferred by chance (Table 1).

Alignment dependence and conformational sensitivity are problems that need to be considered when using Py-CoMFA (3D-QSAR)^50^. In this way, since the Kennard-Stone splitting method using C*sp*^3^ (+ 1) as a probe and STE field produced the most favorable statistical results, we decided to assess the model using docking conformations (Figure S1) in the alignment for QSAR modeling. Despite the model being based on the STE + ELE fields combination presenting great results (Table S2, S3, and Figure S2), the Kennard-Stone splitting method, with C*sp*^*3*^ (+ 1) as the probe and STE field, continues to provide the most satisfactory outcomes for all statistical parameters evaluated.

**Figure 3:**
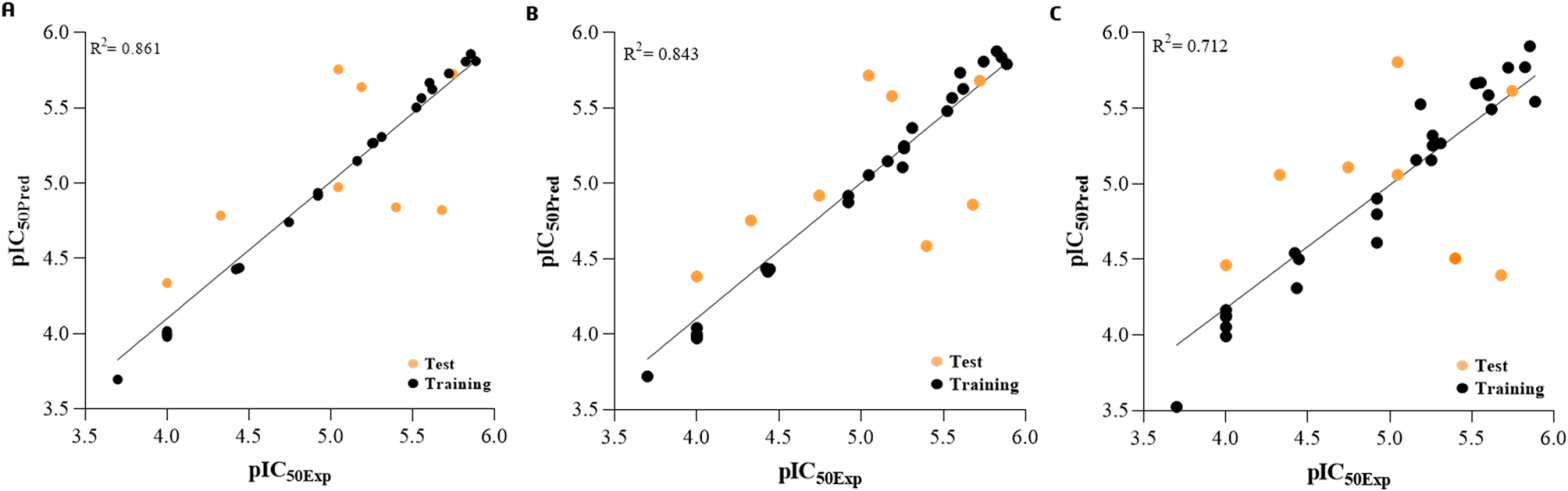
Experimental *versus* predicted pIC_50_ values for the training and test set compounds according to the Py-CoMFA (3D-QSAR) models built using steric field, 2 Å grid spacing, and the Kennard-Stone training and test sets splitting method, by probe atoms and charges: (**A**) C*sp*^3^ (+ 1) (y = 0.9086x + 0.467; R^2^ = 0.8608); (**B**) O*sp*^3^ (− 1) (y = 0.8995x + 0.508; R^2^ = 0.8436); and (**C**) H (+ 1) (y = 0.8144x + 0.9211; R^2^ = 0.7123).

In this way, Fig. 3 shows the plots of the experimental (pIC_50Exp_) and predicted (pIC_50Pred_) biological response values for the training and test sets compounds, according to the Py-CoMFA models built using the STE field, 2 Å grid spacing, and the Kennard-Stone splitting method, showing the best performance for the probe atoms C*sp*^3^ (+ 1) and O*sp*^3^ (− 1) compared to the H (+ 1) probe atom. In fact, the Py-CoMFA models had a squared linear correlation coefficient (R^2^) of 0.861 to C*sp*^3^ (+ 1) (Fig. 3A; y = 0.9086x + 0.467; R2 = 0.8608), 0.843 to O*sp*^3^ (− 1) (Fig. 3B; y = 0.8995x + 0.508; R2 = 0.8436), and 0.712 to H (+ 1) (Fig. 3C; y = 0.8144x + 0.9211; R2 = 0.7123) as probe atoms.

Table 2 presents the predicted pIC_50_ and residual (Res = pIC_50Exp_ − pIC_50Pred_) values calculated with the Py-CoMFA models, considering the Kennard-Stone splitting method, for the training and test set inhibitors during the internal (leave-one-out cross-validation, LOO-cv) and external validations, respectively. Inhibitors of the training and test sets were considered outliers if showing residual values exceeding twice the standard deviation of estimate model (C*sp*^3^, 2*SD = 0.888; O*sp*^3^, 2*SD = 0.948, H, 2*SD = 0.962). Based on this criterion, the Py-CoMFA models did not identify outliers in the training and test sets compounds to C*sp*^3^ and O*sp*^3^ models and just one outlier (**5f**) in the test set of H model (Table 2). The lowest residual values between experimental and predicted pIC_50_ were for the models from C*sp*^3^ (+ 1) and O*sp*^3^ (− 1) and the higher ones for the H (+ 1) atom probe (Table 2). Although the H (+ 1) probe atom model presented the worst performance, the three models are highly correlated, since the residuals of all three models showed a strong Pearson correlation with C*sp*^3^ *versus* O*sp*^3^ (*r* = 0.96), C*sp*^3^ *versus* H (*r* = 0.92), and O*sp*^3^ *versus* H (*r* = 0.92).

**Figure 4:**
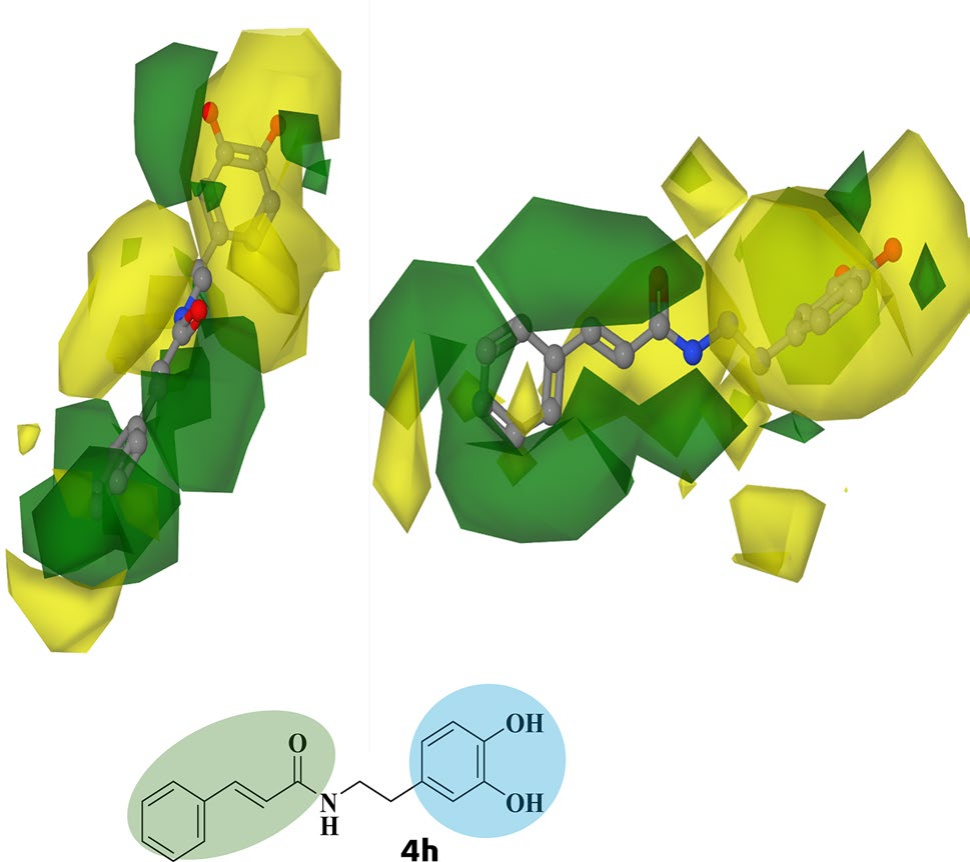
The Py-CoMFA contour maps of steric molecular interaction field (MIF): favorable (green) and unfavorable (yellow) regions for the most active compound **4h**, by probe atoms model C*sp*^3^ (charge =  + 1). Atom colors: carbon (grey), oxygen (red), and nitrogen (blue).

The Py-CoMFA C*sp*^3^ (+ 1) contour maps of the steric MIF of the most active compound **4h** showed favorable (green) fields on the phenyl and amide group, indicating that bulk substituents on those positions could enhance the protein–ligand interactions. However, it is essential to note that substitution in the nitrogen atom of the amide group has unfavorable (yellow) fields, particularly for bulk substituents such as phenyl group (Fig. 4).

**Figure 5:**
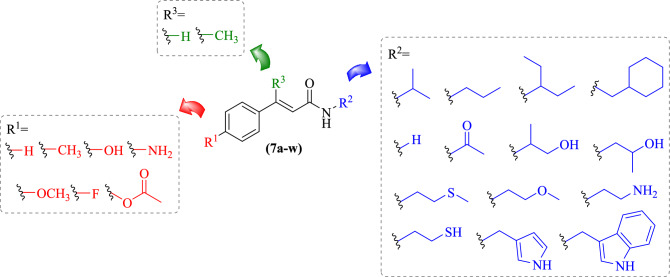
Chemical structures of the proposed cinnamide derivatives (**7–aw**) designed based on the interpretation of the Py-CoMFA (C*sp*^3^, + 1) model to calculate the predict pIC_50_ values.

Based on the information obtained from the contour maps of the best Py-CoMFA predictive model, 23 new compounds (**7a–w**) were designed, maintaining the general cinnamide structure (Fig. 5). In the *para-*position of the phenyl ring, R^1^, we tested substituent groups (R^1^ = H, CH_3_, OH, NH_2_, OCH_3_, F, and O(CO)CH_3_) with different degrees of inductive and/or resonance effect as electron donor or acceptor. As a substituent at the nitrogen atom of the amide group, we tested H, aliphatic groups, with or without heteroatoms, and with cyclohexyl, pyrrole, and indole rings (Fig. 5).

The Py-CoMFA (C*sp*^3^, + 1) model was used to predict the pIC_50_ values of the newly designed compounds (**7a–w**). Out of the 23 compounds analyzed, we selected six that had predicted pIC_50_ values greater than 5.00 M based on the activity of the reference compound **4h** (pIC_50_ = 5.886 M). Although all compounds did not present a predicted biological activity, value greater than the reference compound, interestingly, the highest pIC_50_ values were observed at **7g** (pIC_50Pred_ = 5.169 M) and **7k** (pIC_50Pred_ = 5.169 M) with H at R^1^ position, and indole and cyclohexyl substituents in the R^2^ position, respectively (Table 3), while the pyrrole ring did not contribute to the activity. Among the aliphatic substituents, the best four compounds were **7o**, **7p**, **7q**, and **7b** with acetyl, methyl(thio)ethyl, methoxy-ethyl, and amino groups, respectively. Aliphatic side chains with no heteroatoms or hydroxyl groups did not contribute to the activity as well as *para*-position substitution of the phenyl ring in R^1^, with electron donor or acceptor groups, since pIC_50_ values were lower than unsubstituted derivatives (Table 3).

**Table 3:** Predicted pIC_50_ (M) values for the proposed molecules (**7a–w**) based on the Py-CoMFA model (C*sp*^3^, + 1). In bold, the compounds with pIC_50_ ≥ 5.000 M.

#	Structure	pIC_50Pred_	#	Structure	pIC_50Pred_
7a		4.879	**7m**		4.617
7b		**5.010**	**7n**		4.903
7c		4.946	**7o**		**5.057**
7d		4.996	**7p**		**5.016**
7e		4.849	**7q**		**5.014**
7f.		4.927	**7r**		4.967
7g		**5.169**	**7s**		4.895
7h		3.889	**7t**		4.962
7i		4.994	**7u**		4.915
7j		4.969	**7v**		4.837
7k		**5.151**	**7w**		4.987
7l		4.806			

#### Applicability domain

To assess the applicability domain, we employed two methods: k-nearest neighbors (kNN) using Euclidean distances as metric and Leverage^53–55^. The kNN methodology is centered on assessing data points’ similarity through their feature vectors, with similarity being defined by the distance in the feature space, often computed using the Euclidean distance metric. It specifically recognizes the k-nearest neighbors of a query data point and employs them for predictive purposes or data classification^53^.

In contrast, the leverage technique appraises the impact of individual data points on the dataset’s overall comprehension. It computes leverage metrics for each data point, signifying its influence on the model’s analyses or predictions^53^.

Whereas kNN underscores similarity and neighborhood associations, the leverage approach underscores the significance and sway of individual data points within the dataset. Consequently, while kNN aids in clustering akin data points, the leverage method assists in pinpointing influential or anomalous data points that could considerably impact model performance or comprehension.

**Figure 6:**
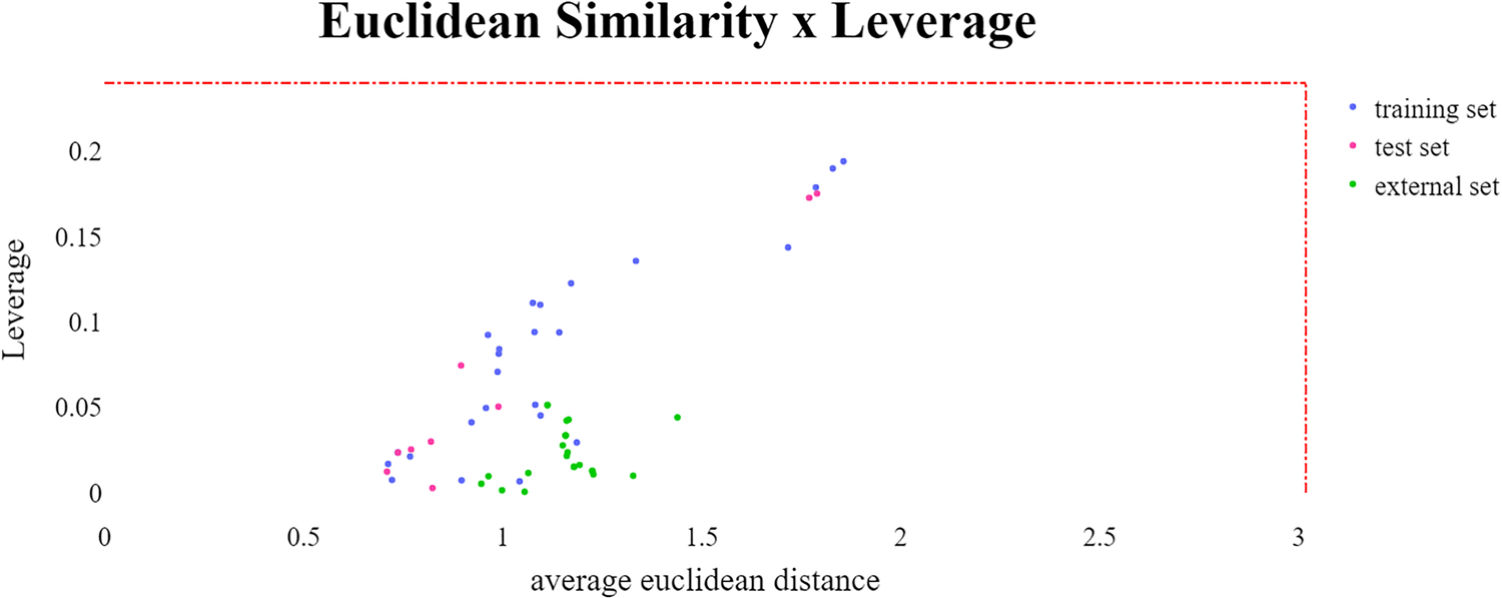
Applicability domain Euclidean *vs* Leverage plot to Py-CoMFA model.

Upon evaluating the limits established by kNN (3.02) and Leverage (0.24) for the training set, we found no outlier compounds in the external set, indicating that its chemical space aligns closely with the training set’s (Fig. 6)^56,57^.

### Molecular docking

The constructed Py-CoMFA (3D-QSAR) model was based on compound **4h** as an alignment reference. This compound has been reported in the literature for its mixed-type inhibition^4^, which means it can bind to allosteric sites both to the free enzyme or enzyme–substrate complex. On the other hand, compound 4e is reported as a competitive inhibitor ^4^ binding to the active site of the free enzyme. As compounds **7g** and **7k** share a similar structure with the reference compound and were chosen due to their predicted pIC_50_ activity, we aimed to investigate their behavior in both the potential allosteric sites and the active site of the LaARG protein. Our goal was to compare their behavior with the reference inhibitors **4e** and **4h**, respectively.

**Figure 7:**
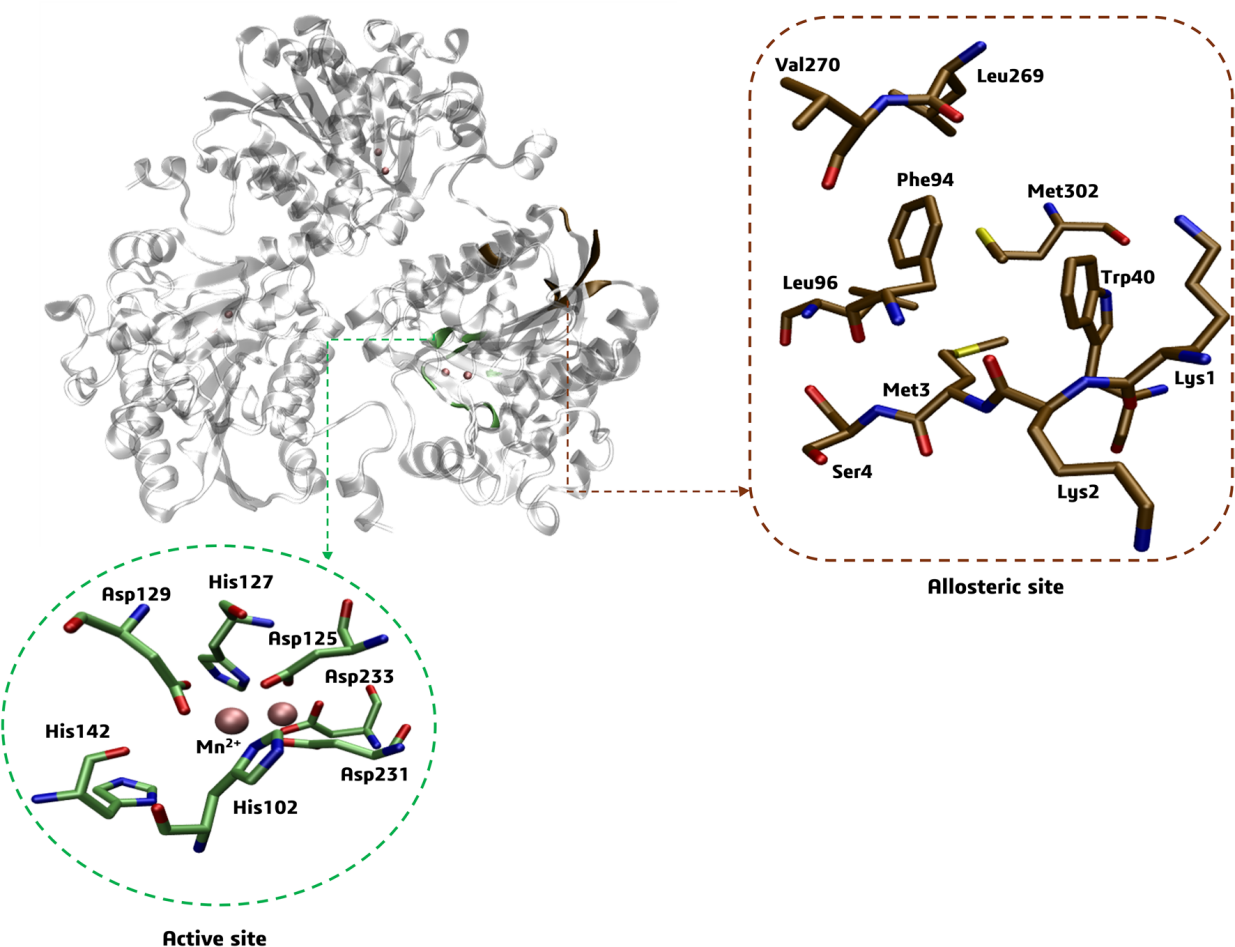
The active site (green color) and the putative allosteric site (ochre color) of the *La*ARG model were detected using the FTMap server (expanded for a monomer).

The FTMap^58^ server was used to detect hot spot residues and identify potential drug-binding sites, including the active site, resulting in two clusters of interest (for more details, please refer to the Supporting Information). The first cluster corresponds to the active site region (amino acid residues detected as hot spots: His102, His127, Asp125, Asp129, His142, Asp231, and Asp233), including two Mn(II) cations, which are presented in a zoom view within a green color (Fig. 6). The second cluster corresponds to an allosteric site region (amino acid residues detected as hot spots: Lys1, Lys2, Met3, Ser4, Trp40, Phe94, Leu96, Leu269, Val270, and Met302), which is presented in a zoom view within an ochre color (Fig. 7). This allosteric site corresponds to a cavity composed of residues from one of the monomers close to the protein–protein interface and in a region opposite the catalytic site.

**Figure 8:**
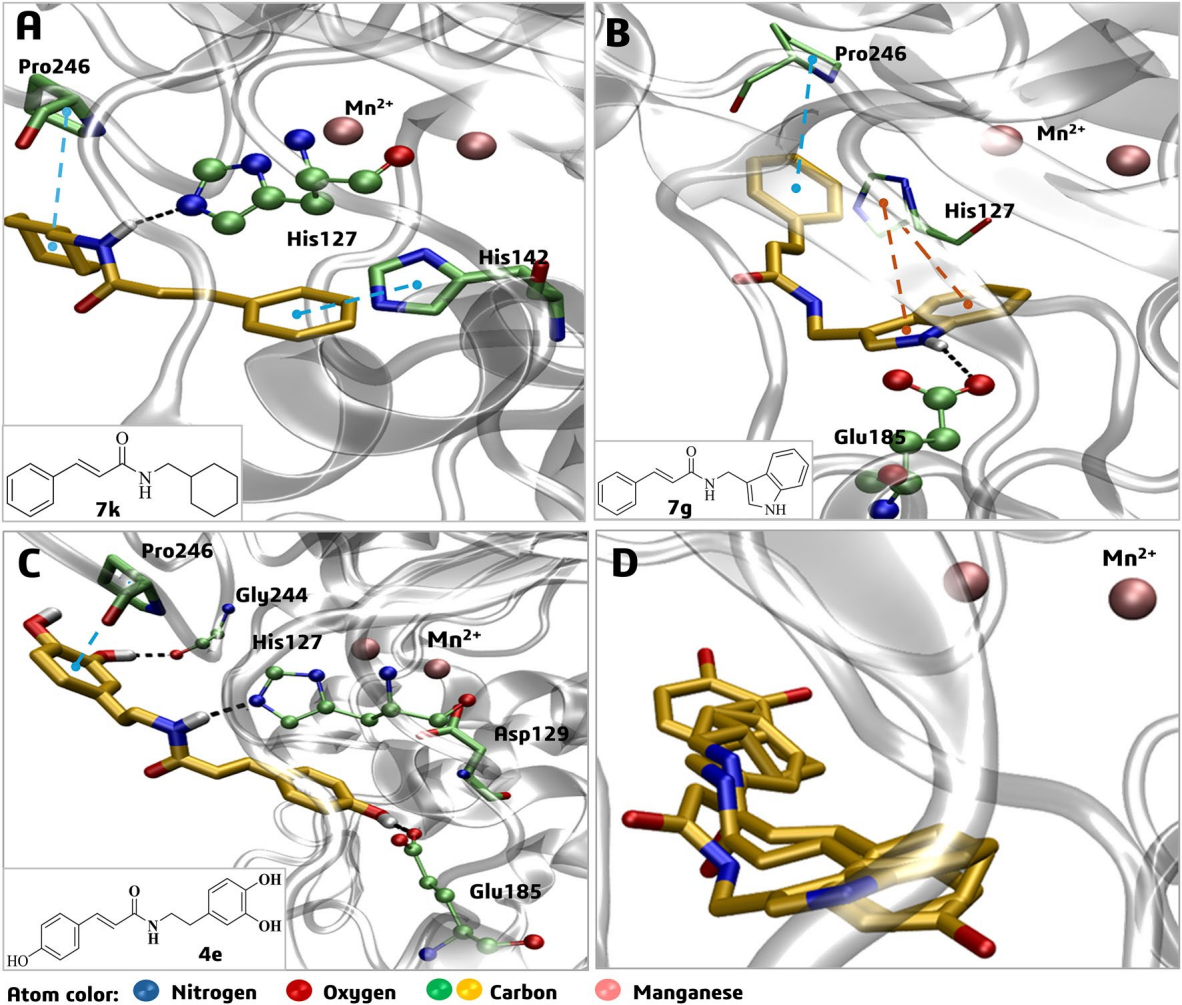
Best molecular docking poses of cinnamide derivatives **7g** (**A**) and **7k **(**B**) and reference **4e **(**C**) within the active site of the *La*ARG model and the overlapped poses of **7g**, **7k** and **4e** (**D**). The dashed black lines represent the H-bond interactions with amino acids (represented by spheres). Residues are represented by sticks (green color) interacting with pi-alkyl (dashed yellow lines), pi-pi T-shaped (dashed orange lines), or alkyl-alkyl (dashed blue lines).

In the first instance, we investigated the potential interaction of derivatives **7g** and **7k** at the enzyme’s active site by molecular docking. As previously mentioned, compound **4e** is described as a competitive inhibitor ^4^, so both compounds, **7g** and **7k**, were docked in the first cluster corresponding to the active site of the *La*ARG model (Fig. 8). In the best docking pose of **7g** within the *La*ARG active site (Fig. 8A), a strong charge-assisted H-bond interaction is observed between the NH group (neutral donor) of the indole ring of **7g** and the carboxylate group (ionized acceptor) of Glu185, while this aromatic indole ring also performs a pi-pi T-shaped interaction with the aromatic imidazole ring of His127. Meanwhile a pi-alkyl interaction is observed between the phenyl ring of the cinnamide moiety of **7g** and Pro246. Likewise, in the best docking pose of **7k** within the *La*ARG active site (Fig. 8B), an H-bond interaction is observed between the cinnamide NH group and the N3 atom of the imidazole ring of His127, while the cinnamide phenyl ring performs a pi–pi interaction with the imidazole ring of His142, as well as cyclohexyl methyl substituent at the R^2^ position performs an alkyl-alkyl interaction with Pro246 (Fig. 8B). The inhibitor **4e** had a *p*-OH substituent at the R^1^ position. This allowed for a H-bond interaction with Glu185. Two more interactions were observed with the amino acids His127 and Gly244. Similar to compounds **7g** and **7k**, there was also an interaction between Pro246 and the 3,4-dihydroxyphenyl ring (Fig. 8C).

Regardless of the types of interactions of these compounds, by the rings as substituents or with the cinnamide moiety, it is worth highlighting that compounds **7g** and **7k** showed the best-predicted pIC_50_ values of 5.169 M and 5.151 M, respectively. These compounds occupy the same region within the active site at approximately 6.6 (**7g**) and 7.8 Å (**7k**) from Mn^2+^ ions, interacting mainly with the same His127 and Pro246 residues, presenting only an inverted head-to-tail orientation in relation to each other, in addition to a similar binding mode compared to reference inhibitor **4e** (Fig. 8D). Those interactions were important for the ligand binding on the enzyme’s active site, which may reflect in the inhibition of the arginase enzyme.

**Figure 9:**
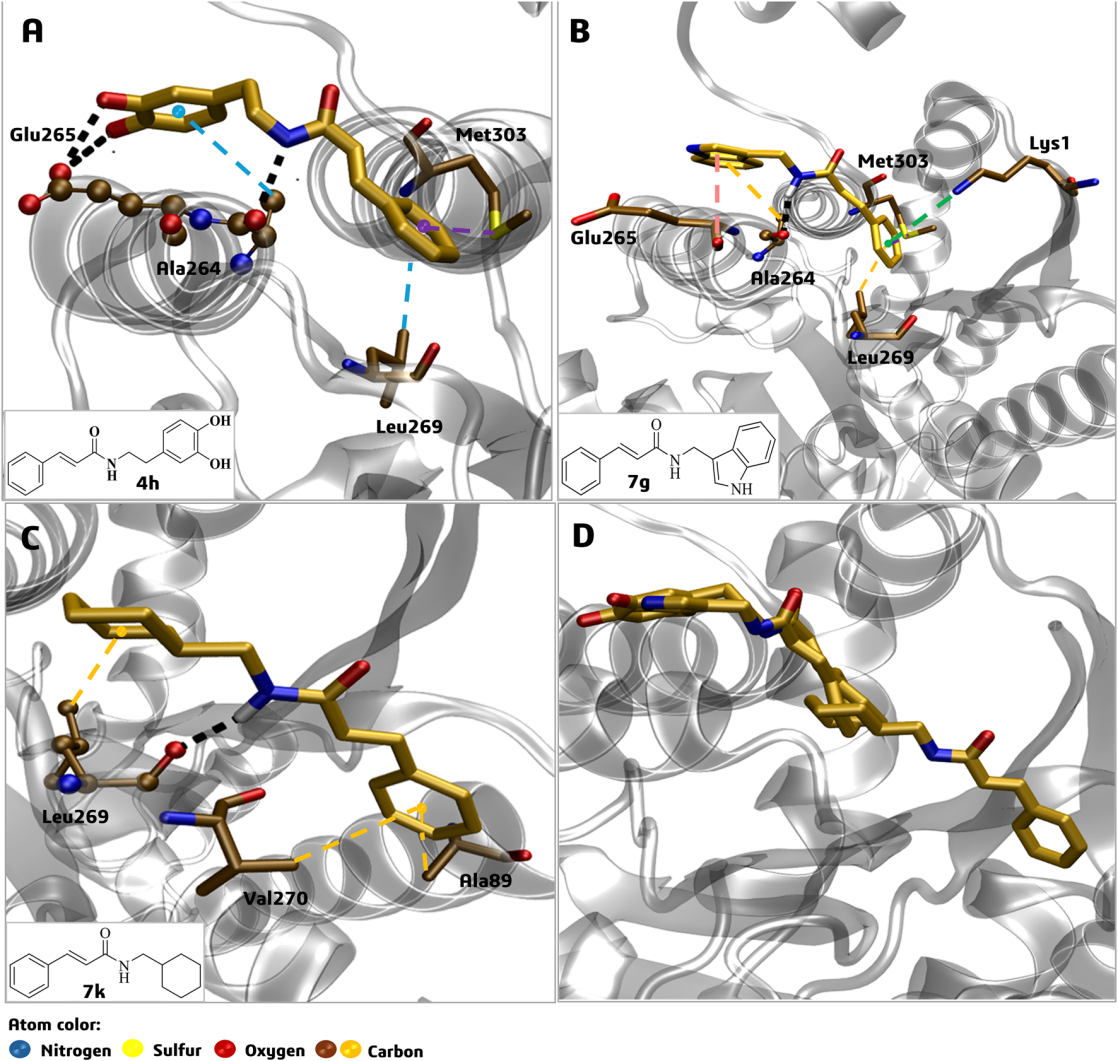
Best molecular docking poses of cinnamide derivatives **4h **(**A**), **7g** (**B**), and **7k** (**C**) within an allosteric site of the *La*ARG model and the overlapped poses of **4h**, **7g**, and **7k** (**D**). The dashed black lines represent the H-bond interactions with amino acids (represented by spheres). Residues represented by sticks (ochre color) interacting by pi-alkyl (dashed yellow lines), pi-sulfur (dashed purple lines), cation-pi (dashed green lines), or amide-pi stacked (dashed pink lines).

Afterward, we investigated the potential interaction of compounds **4h**, **7g**, and **7k** into the second cluster of *La*ARG, corresponding to an allosteric site, by molecular docking (Fig. 9). In the best docking pose of **4h** within the *La*ARG allosteric site (Fig. 9A), H-bond interactions are observed between the cinnamide NH group and the carbonyl oxygen atom of the Ala264 main chain, while the cinnamide phenyl ring performs pi-alkyl, and pi-sulfur interactions with the side chain groups of Leu269 and Met303, respectively. In addition, the 3,4-dihydroxyphenyl ring of **4h** performs H-bond interactions with the oxygen atom of Glu265 (Fig. 9A).

In the best docking pose of **7g** (Fig. 9B), an H-bond interaction is observed between the cinnamide NH group and the carbonyl oxygen atom of the Ala264 main chain likewise **4h**. The cinnamide phenyl ring performs cation-pi, pi-alkyl, and pi-sulfur interactions with the side chain groups of Lys1, Leu269, and Met303, respectively. In addition, the indole ring of **7g** performs two interactions, an amide-pi stacking with Glu265 and a pi-alkyl with Ala264 (Fig. 9B).

In the best docking pose of **7k** (Fig. 9C) there is an H-bond interaction between the cinnamide NH group and Leu269, while the cinnamide phenyl ring performs two pi-alkyl interactions with the side chains of Ala89 and Val270 (Fig. 9C). In general, compounds **4h** and **7g** bound similarly (Fig. 9D) to the allosteric site, while **7k** bound closer to the protein–protein interface of the enzyme trimer, i.e., between two monomers.

### Molecular dynamics simulations

Subsequently, we conducted classical molecular dynamics (MD) simulations in aqueous systems with the best docking poses of **4h** within the allosteric site, **4e** within active site, and **7g** and **7k** within both active and allosteric sites of the *La*ARG model to verify the dynamic behavior of the putative interaction modes and binding affinities of those derivatives during 200 ns, using the GROMACS software^59^ with Charmm36 force field^60^.

#### Molecular dynamics simulations in the active site

**Figure 10:**
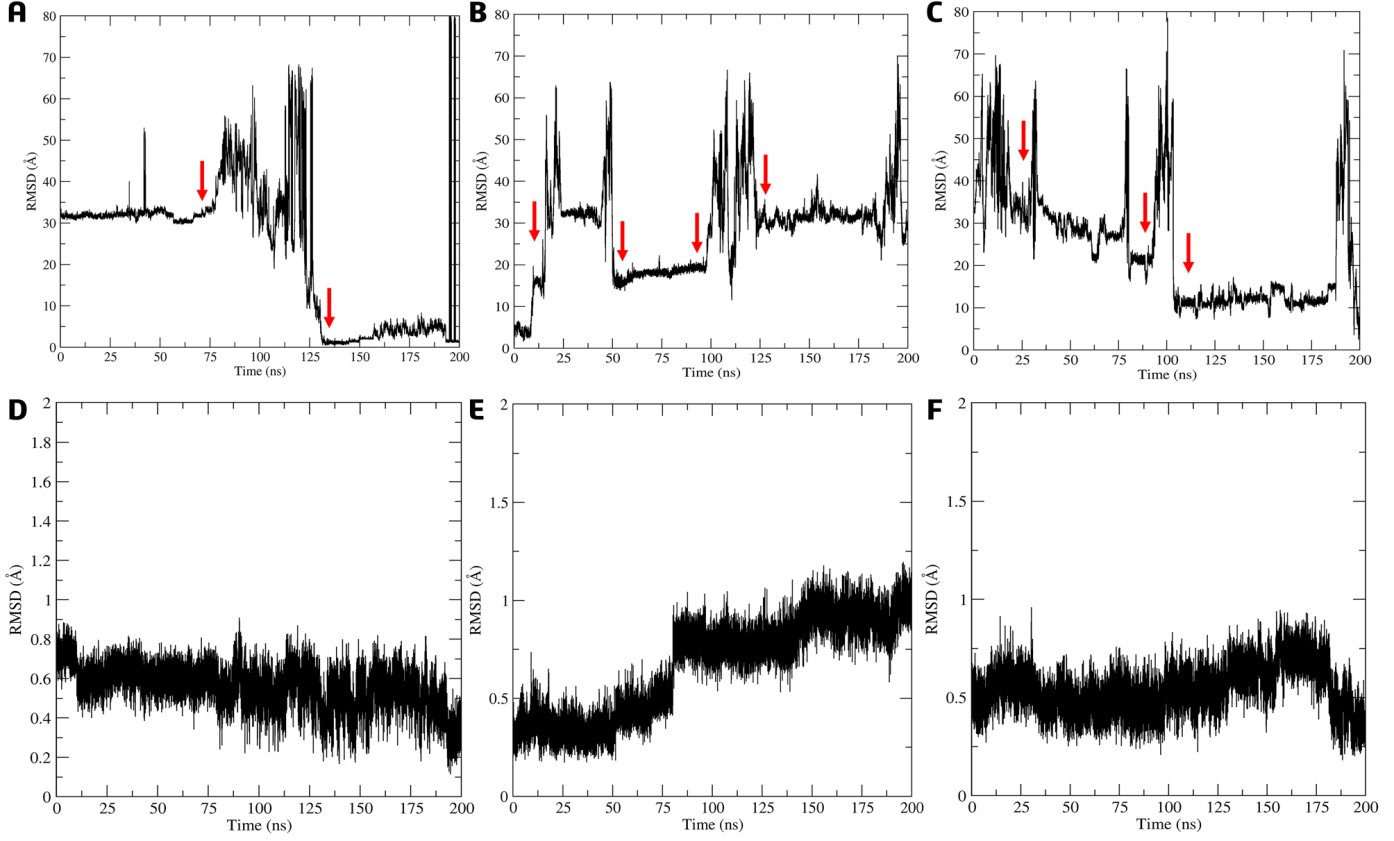
RMSD (Å) analysis relative to the atoms of **4e** (**A**), **7g** (**B**), and **7k** (**C**) and Cα-RMSD analysis of the amino acid relative to complex with **4e** (**D**), **7g** (**E**), and **7k** (**F**) in the *La*ARG model by molecular dynamics simulations in aqueous systems (200 ns), considering the initial ligand positions within the active site. The red arrow highlights the ligand movement.

The stability and persistence of interactions were evaluated by RMSD (root-mean-square deviations) of the ligand atoms and Cα atoms of the amino acids in the protein–ligand complexes throughout the MD simulations (Fig. 10A–C). During the RMSD ligand atoms analysis of simulation conducted on the active site, it was observed the reference inhibitor **4e** moved out of the active site within the first nanoseconds of the MD (Fig. 10A). It then exhibited movement between the 75 and 125 ns, before returning to its initial position and binding to the active site in the final phase of the simulation. This resulted in a high value of RMSD and standard deviation (SD) of 23.30 ± 16.7 Å. Compound **7g**, on the other hand, showed a tendency to move away after 10 ns, bound to the surface of the allosteric site from 50 to 100 ns before leaving again (Fig. 10B). The mean RMSD value was 28.4 ± 10.6 Å and similar to **4e**. Compound **7k** was observed to leave the active site at the beginning of the simulation, presenting the highest RMSD and SD values of 30.4 ± 19.7 Å (Fig. 10C). We analyzed the Cα-RMSD of the amino acids from the active site of the *La*ARG-ligand complexes. To ligands **4e**, **7g** and **7k** bonded in the active site, Cα-RMSD values and SD were low, 0.56 ± 0.11 Å, 0.54 ± 0.12 Å and 0.67 ± 0.25 Å, respectively (Fig. 10D–F).

**Figure 11:**
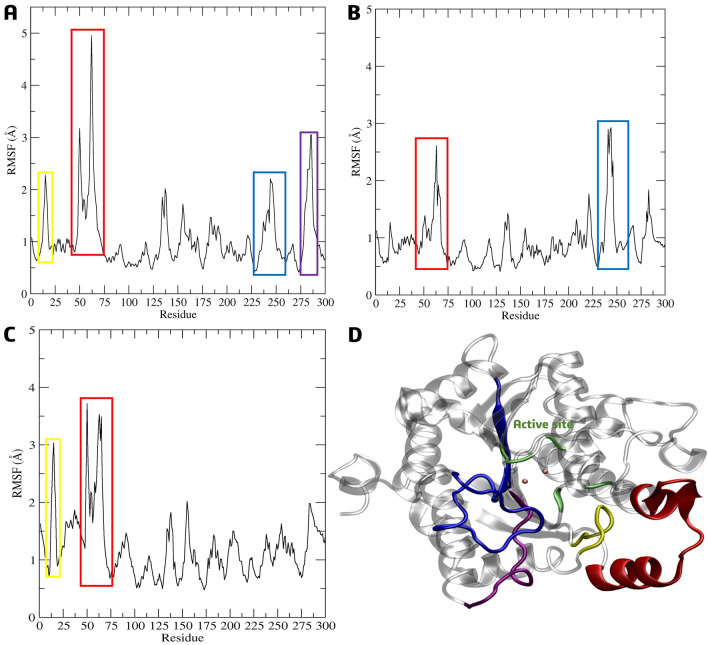
Cα-RMSF analysis relative to the molecular dynamics simulations in aqueous systems (200 ns) of the *La*ARG-ligand complexes of **4e **(**A**), **7g **(**B**) and **7k **(**C**) in the active site; (**D**) 3D-structure of the *La*ARG model highlighting the active site (green) and residues groups purple, yellow, blue, and red with the highest RMSF values.

The root-mean-square fluctuation (RMSF) during the 200 ns of simulation was used to evaluate the residue’s mobility. The *La*ARG model residues with the highest observed RMSF values were grouped into four main groups (Fig. 11D). The first group comprises the residues Gln14-Tyr25 (colored in yellow, Fig. 11A–D), which are located around the active site (in green, Fig. 11D), approximately 10 Å from the two Mn(II) cations, and presented RMSF values of 2.3 to 3.0 Å. The second group comprises the residues Gly50-Ala75 (in red, Fig. 11A–D), which are close to the protein surface and their RMSF values varied from 3.72 to 4.9 Å. The third group comprises the residues Pro225-Gly250 (in blue, Fig. 11A,B,D), which are located next to the active site and present RMSF values between 2.0 and 2.9 Å. The fourth group is related to residues Val275-His287 (in purple, Fig. 11A and D), which is below the active site, with considerable RMSF values of around 3.0 Å. It was evident that the greatest fluctuations contribution was from the **4e** inhibitor, despite the planned compounds **7g** and **7k** also showing similar values in the surrounding residues near the active site.

**Figure 12:**
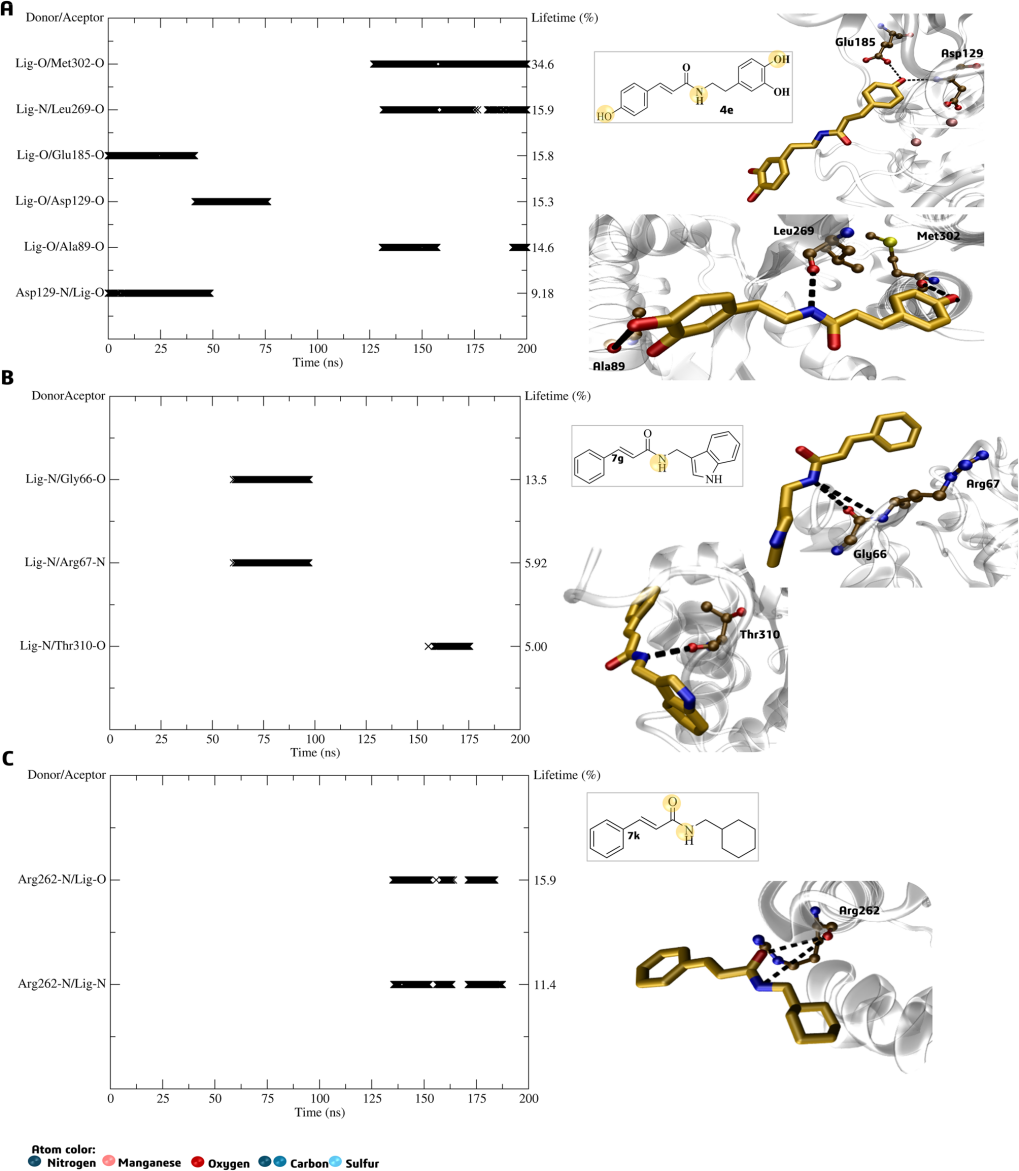
Active site H-bond lifetime (%) and representative interactions of (**A**) *La*ARG-**4e, **(**B**) *La*ARG-**7g** and (**C**) *La*ARG-**7k** complexes. The yellow circle in the 2D structure highlights the atoms responsible for the interactions.

The analysis of inhibitor **4e**’s H-bond interactions revealed that it has the potential to inhibit the target protein by interacting with specific residues such as Asp129, Glu185, Leu269, and Met202 present in the active site (Fig. 12A). The lifetime of these interactions ranged from 9.18% to 34.6% (Fig. 12A), although the RMSD analysis pointed out a lack of interactions between the period of 75 to 125 ns. The H-bond analysis in the active site of the *La*ARG-**7g** and *La*ARG-7**k** complexes revealed that these intermolecular interactions involving the ligands and certain residues, such as Gly66 and Thr310 (as shown in Fig. 12B) and Arg262 (Fig. 12C), had a short lifetime (5% to 15.9%) and were located away from the initial binding poses (active site), as pointed out by RMSD analysis.

We calculated the binding free energy (ΔG_bind_), using the MM-PBSA method, for ligands that remained bound to the active site of the La*ARG* model during the MD simulations. The ΔG_bind_ values reflect that **4e** (− 17.8 kcal/mol) and **7g** (− 20.6 kcal/mol) presented similar affinity for the active site (Table 4). Upon analysis, we also observed that the van der Waals and solvent-accessible surface values for cinnamates **7g** and **7k** are quite similar. However, we noticed that the difference in ΔG_bind_ values can be attributed to the higher solvation cost of **7k**, which has substantially higher values of 15.1 kcal/mol when compared to **7 g** with its lower value of 5.14 kcal/mol. This clearly indicates the higher ΔG_bind_ value observed (− 7.49 kcal/mol).

**Table 4:** The binding free energy (ΔG_bind_) terms of the *La*ARG-ligand complexes, considering the active site, calculated for **4e**, **7g**, and **7k** with the MM-PBSA method (mean ± standard deviation energies; kcal/mol): van der Waals (ΔE_vdW_), electrostatic (ΔE_elect_), solvation (ΔE_solv_), and solvent accessible surface area (ΔE_sasa_).

Ligand	ΔE_vdw_	ΔE_elect_	ΔE_solv_	ΔE_sasa_	ΔG_bind_
	− 30.0 ± 1.67	− 19.2 ± 1.57	34.9 ± 2.50	− 3.46 ± 0.12	− 17.8 ± 2.18
	− 17.1 ± 1.63	− 6.46 ± 1.45	5.14 ± 1.17	− 2.26 ± 0.13	− 20.6 ± 2.11
	− 17.3 ± 1.26	− 2.99 ± 1.10	15.1 ± 2.70	− 2.30 ± 0.16	− 7.49 ± 2.21

#### Molecular dynamics simulations in allosteric site

**Figure 13:**
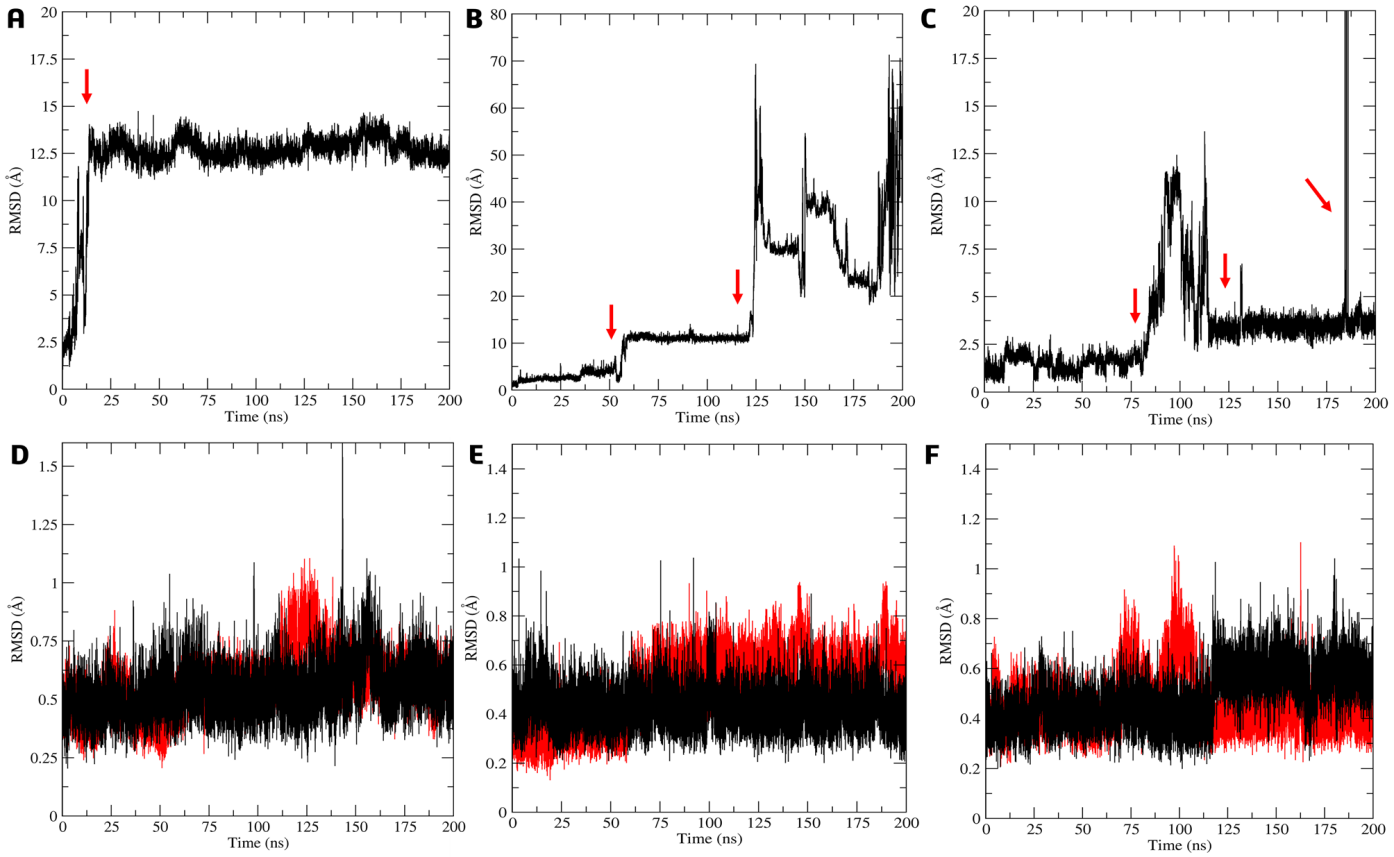
RMSD (Å) analysis relative to the atoms of **4h** (**A**), **7g** (**B**), and **7k** (**C**) and Cα-RMSD analysis of the amino acid relative to complex with **4h** (**D**), **7g** (**E**), and **7k** (**F**) in the *La*ARG model by molecular dynamics simulations in aqueous systems (200 ns), considering the initial ligand positions within the allosteric (black) or active (red) site. The red arrow highlights the ligand movement.

Subsequently, we conducted MD simulations in aqueous systems with the best docking poses of **4h**, **7g,** and **7k** within the allosteric site of the *La*ARG model. During the simulation, the compound **4 h** displayed notable movement in the allosteric site after 10 ns and maintained its position throughout the rest of the simulation, with an RMSD value of 12.2 ± 2.07 Å, indicating a significant shift in its conformation (Fig. 13A).

On the other hand, RMSD analysis of **7g** (Fig. 13B) indicated the persistence on the binding site until 125 ns of the simulation, showing slight movement between 50 and 125 ns inside the cavity before eventually dissociating with RMSD = 17.28 ± 14.13 Å. Like reference **4h**, ligand **7k** was found to have a strong affinity for the allosteric site (Fig. 13C) since the binding persisted almost throughout the entire period of the simulations, with only two slight movements observed between 80 and 112 ns and 185 ns, while the RMSD and SD values were low (4.34 ± 0.76 Å).

When ligands **4h**, **7g**, and **7k** were bonded on the allosteric site, the Cα-RMSD to the active site were lower, 0.57 ± 0.13, 0.51 ± 0.14, 0.45 ± 0.10, Å, respectively (in red, Fig. 13D–F). Analysis of the Cα-RMSD for the allosteric site also indicated low variation of 0.54 ± 0.11 Å (**4h**), 0.43 ± 0.09 Å (**7g**), and 0.49 ± 0.12 Å (**7k**), suggesting no significant shift compared to the docking complex (in black, Fig. 13D–F). The structures were stabilized after the first nanoseconds of simulation with a standard deviation below 0.14 Å (Fig. 13D–F).

**Figure 14:**
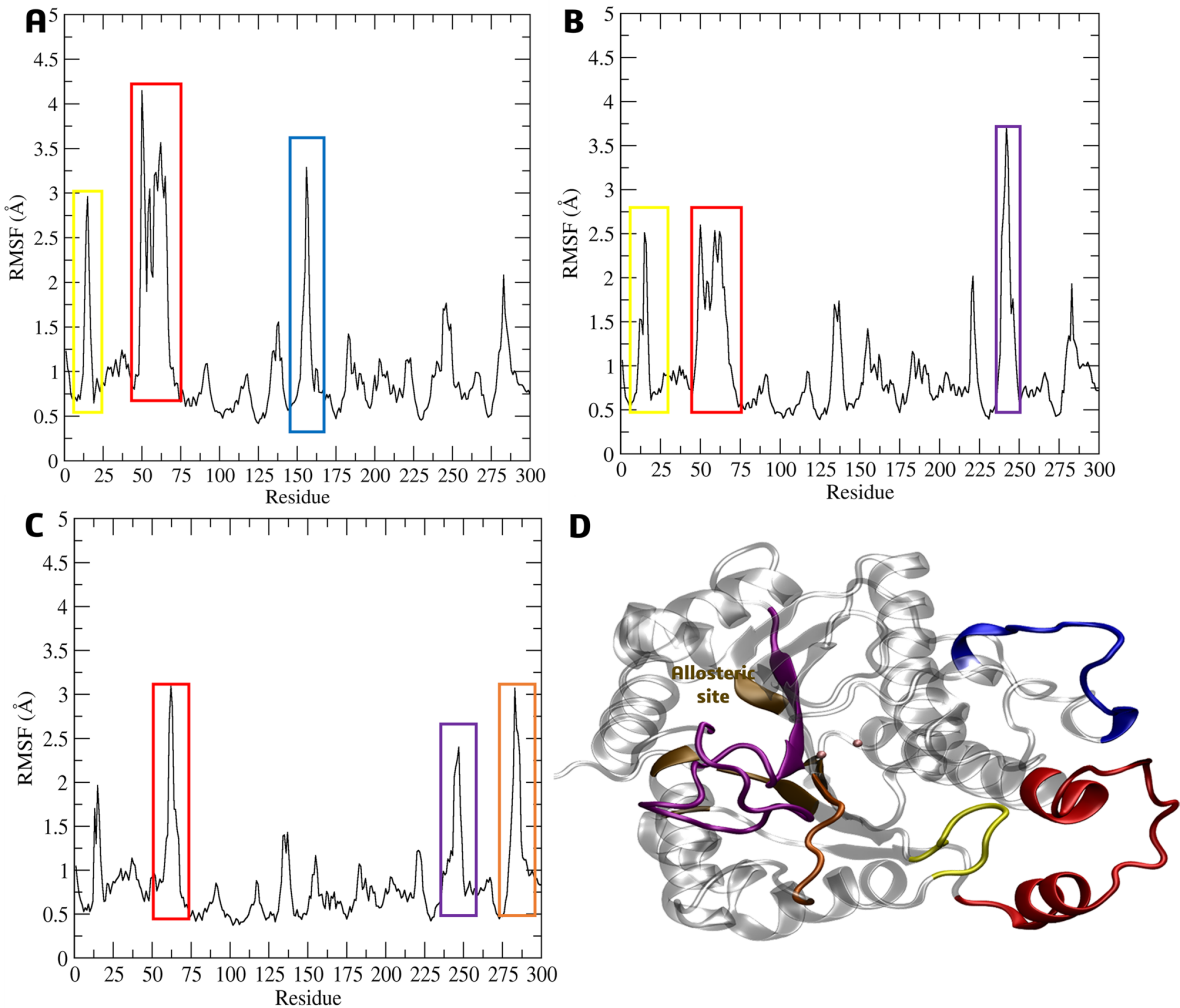
Cα-RMSF analysis relative to the molecular dynamics simulations in aqueous systems (200 ns) of the *La*ARG-ligand complexes of **4h **(**A**), **7g **(**B**) and **7k **(**C**) in the allosteric site; (**D**) 3D-structure of the *La*ARG model highlighting the allosteric site (ochre) and residues groups purple, yellow, blue, orange, and red with the highest RMSF values.

As observed in the Cα-RMSF analysis of the compounds in the active site, their presence in the allosteric site also caused fluctuations in the regions, mainly of protein loops, to residues Gln14-Tyr25 (colored in yellow, Fig. 14A,B,D) and Gly50-Ala75 (in red, Fig. 14A–D) closed to the protein surface, and Pro225-Gly250 next to allosteric site (in purple, Fig. 14B–D), ranging from 2.5 to 4.2 Å. In addition to these, two more regions presented high RMSF values, belonging to residues Leu150-Pro170 (in blue, Fig. 14A–D) and Val275-Arg300 (in orange, Fig. 14C–D) about 3.0 Å.

**Figure 15:**
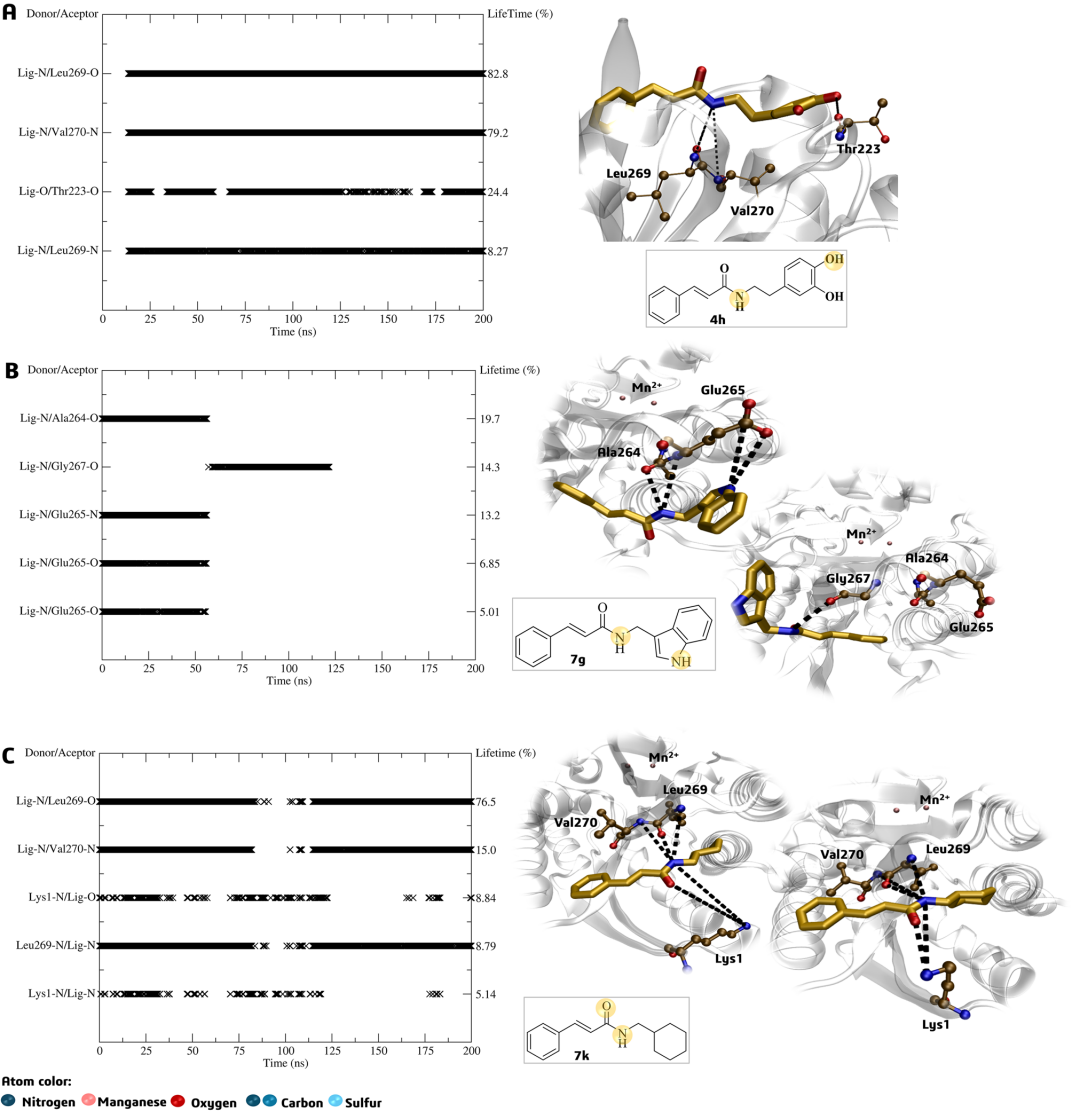
Allosteric site H-bond lifetime (%) and representative interactions of *La*ARG-**4h **(**A**) *La*ARG-**7g **(**B**), and *La*ARG-**7k **(**C**) complexes. The yellow circle in the 2D structure highlights the atoms responsible for the interactions.

About H-bond interactions observed in the allosteric site, the reference inhibitor **4h** showed persistent interaction with residues Leu269 and Val270 with high lifetime values of 79.2% (and 8.27%) and 82.8%, respectively. In addition, also performs H-bond with Thr233 (24.4%) continually throughout the MD simulation (Fig. 15A). The derivative **7g** interacted with residues Ala264 and Glu265 within the first 50 ns and a 5–19% lifetime. It also interacted with Gly267 from 50 to 125 ns, with a lifetime of 14%, before disconnecting from the enzyme (Fig. 15B). Notably, in arginase from *Leishmania mexicana*, a salt-bridge cluster (Glu277-Arg216) is known to stabilize the formation of the trimer, which is not present in the human arginase I^61^. In the LaARG, this salt-bridge is conserved by Glu265-Arg204 and could lead to a possible selectivity for the parasite enzyme. Despite their short lifetimes, these interactions at the allosteric site were equally persistent than those observed at the active site, suggesting that **7g** may have affinity for both sites. On the other hand, **7k** showed high affinity for *La*ARG’s allosteric site, presenting lifetime values of up to 76% throughout the MD simulation, mainly with residue Leu269, as reference inhibitor **4h**, and others, such as Lys1 and Val270 (Fig. 15C).

Finally, we calculated ΔG_bind_ for ligands that remained bound to the allosteric (**4h**, **7g**, and **7h**) site of the La*ARG* model during the MD simulations (Table 5). It is more evident that cinnamide derivative **7k** is promising for inhibition of *La*ARG in the allosteric site since it presented an equivalent ΔG_bind_ value of − 16.6 ± 1.76 kcal/mol as reference inhibitor **4h** (− 17.0 ± 2.14 kcal/mol), while derivative **7g** showed lower ΔG_bind_ =  − 13.4 ± 1.95 kcal/mol.

**Table 5:** The binding free energy (ΔG_bind_) terms of the *La*ARG-ligand complexes, considering the allosteric site, calculated for **4h**, **7g**, and **7k** with the MM-PBSA method (mean ± standard deviation energies; kcal/mol): van der Waals (ΔE_vdW_), electrostatic (ΔE_elect_), solvation (ΔE_solv_), and solvent accessible surface area (ΔE_sasa_).

Ligand	ΔE_vdw_	ΔE_elect_	ΔE_solv_	ΔE_sasa_	ΔG_bind_
	− 27.9 ± 1.41	− 6.00 ± 2.09	20.1 ± 2.08	− 3.13 ± 0.14	− 17.0 ± 2.14
	− 16.5 ± 1.57	− 0.28 ± 0.56	5.62 ± 1.95	− 2.23 ± 0.15	− 13.4 ± 1.95
	− 27.7 ± 1.75	− 2.89 ± 0.67	17.3 ± 1.08	− 3.11 ± 0.18	− 16.6 ± 1.76

Based on our previous discussions, the binding free energy values also indicate the favorable hydrophobicity contribution of phenyl and cyclohexyl groups as substituents, probably due to the characteristic of several residues on the *La*ARG allosteric site, such as valine, leucine, phenylalanine, serine, methionine, and tryptophan. As a result, the **7k** derivative with phenyl and cyclohexyl have stronger interactions within this pocket also due to molecular volume very similar to **4h** than the indole-substituted **7g,** which is larger.

Finally, the highest energy cost of desolvation of the binding site was observed for cinnamides **4h** (20.1 kcal/mol) and **7k** (17.3 kcal/mol) due to their substituents characteristics when compared to the indole-substituted **7g** (5.62 kcal/mol), in addition to highest energy values of the van der Waals (**4h**, − 27.9 kcal/mol, and **7k**, − 27.7 kcal/mol) and electrostatic terms (**4h**, − 6.00 kcal/mol, and **7k**, − 2.89 kcal/mol) required for a favorable interaction and resulting in their best ΔG_bind_ values (Table 5). It was noted that ΔG_bind_ analysis of the ligands was consistent, as previously emphasized by RMSD, RMSF, and intermolecular interactions via H-bonding, demonstrating the proposed cinnamide **7k** promising as a mix or uncompetitive *La*ARG inhibitor.

In conclusion, it is worth noting that the H-bond interactions between the *La*ARG model and cinnamide derivatives (**4e**, **4h**, **7g**, **7k**) occur through the amide group, which acts as a pharmacophore feature. However, the phenyl and cyclohexyl rings of proposed cinnamide **7k** are crucial for interaction in the allosteric site, which is mostly made up of residues with aliphatic side chains and is more hydrophobic compared to the active site formed by aspartate and histidine residues and more adaptable to interactions with indole ring of the **7g**. It is crucial to emphasize that these compounds should be synthesized and studied in vitro against the arginase molecular target. This approach is necessary to obtain precise and confirmatory details about their inhibitory mechanism of action.

### Drug-likeness (oral bioavailability) and ADMET (absorption, distribution, metabolism, excretion, and toxicity) in silico predictions

After analyzing the interactions evidenced by the molecular docking and molecular dynamics results, the two promising compounds, **7g** and **7k**, had some of their properties related to drug-likeness (oral bioavailability) and ADMET (absorption, distribution, metabolism, excretion, and toxicity) profile predicted in silico (Table 6).

**Table 6:** In silico predicted properties related to drug-likeness (oral bioavailability) and ADMET (absorption, distribution, metabolism, excretion, and toxicity) profile of the promising compounds **7g** and **7k**.

Class	Properties	**7g**	**7k**
Drug-likeness (physicochemical properties related to oral bioavailability)	MW	276.130	243.16
	LogP	3.299	4.114
	HBA	3	2
	HBD	2	1
	RotB	5	5
	TPSA	44.89	29.1
	Num. Viol	0	0
	LogS	− 4.233	− 4.647
Absorption	GI abs	High	High
	P-gp inhib/subs	Yes/No	Yes/No
Distribution	BBB perm	Yes	Yes
Metabolism	CYP1A2	Inhibitor	Inhibitor
	CYPC19	Inhibitor	Inhibitor
	CYP2C9	No	No
	CYP2D6	Inhibitor	No
	CYP3A4	Inhibitor	No
Excretion	t½	Short	Short
Toxicity	Mutagenic	No	No
	Tumorogenic	No	No
	Irritant	No	No
	Reproductive	No	No
	Hepatotoxicity	No	No
	hERG blockers	No	No

MW = molecular weight (g mol^−1^); LogP = Log (base 10) of the partition coefficient (P) of a solute between 1-octanol and water at pH = 7; HBA = number of H-bond acceptors (sum of N + O); HBD = number of H-bond donors (sum of NH + OH); RotB = number of rotatable bonds; TPSA = topological polar surface area (Å^2^); Num. Viol. = number of violations of the rules of Lipinski and Veber; LogS = Log (base 10) of the solubility (S) of a solute in water (mol L^−1^); GI abs = Gastrointestinal absorption; BBB perm. = Blood–brain barrier permeability; P-gp inhib/subs = P-glycoprotein (efflux pump) inhibitor or substrate; CYP (1A2, C19, 2C9, 2D6, 3A4) = activity on cytochrome P-450 (CYP) enzymes; t½ = half-life; Reproductive = Reproductive effect.

In our in silico study, according to the drug-likeness predicted properties (MW ≤ 500; LogP ≤ 5; HBA ≤ 10; HBD ≤ 5; TPSA ≤ 140) (Table 6), we have found that **7g** and **7k** do not violate any of the Lipinski and Veber rules. Moreover, both compounds have the potential to be promising as they show predicted high absorption from the gastrointestinal (GI) tract (Table 6). This indicates that they can cross the cell barriers and enter the bloodstream to reach their biological target^62^. However, it is important to note that these compounds also have the potential to cross the blood–brain barrier (BBB), which may cause toxic side effects^62^ (Table 6).

Compounds **7g** and **7k** were predicted as non-substrate of P-glycoprotein (P-gp) (Table 6), an efflux pump protein that impacts drug pharmacokinetics by carrying substances out of cells^63^. Therefore, **7g** and **7k** should not affect P-gp function, avoiding overdoses in combined therapies (Table 6).

Cytochrome P450 (CYP) enzymes are fundamental in the metabolism of drugs and xenobiotics^64^. According to our in silico predictions, **7g** and **7k** may have the ability to inhibit CYP1A2 and CYPC19. Additionally, **7g** may also inhibit CYP2D6 and CYP3A4. Since the CYP1 enzyme family is responsible for metabolizing carcinogenic substances, we should be cautious about administering any substances that inhibit this enzyme^65^. In addition, the two compounds showed probability of having short half-life (Table 6).

Regarding toxicity predictions, the two compounds did not show any evidence of mutagenic, tumorigenic, reproductive, or irritant properties. They also did not demonstrate a risk of hepatotoxicity. Additionally, there was no indication that they would be active as hERG blockers, which could potentially induce lethal arrhythmia^66^ (Table 6).

## Materials and methods

### Data collection and chemical structure construction

A set of 34 inhibitors of the *Leishmania (L.) amazonensis* arginase (*La*ARG) from different chemical classes and the corresponding half-maximal inhibitory concentration (IC_50_, μM) values were collected from data published in the literature covering the years from 2018 to 2023 and selecting active compounds with no reported in silico studies. These 34 inhibitors belong to the chemical classes of chromones (**1a**–**1d**)^67,68^, pyrazolo-pyrimidines (**2a–2f**)^44^, phenylhydrazines (**3a**–**3f**)^45^, cinnamides (cinnamic acid amides or cinnamides) (**4a–4k**)^4^, and esters (**5a**–**5f**)^46^ derived from 3,4-dihydroxycinnamic acid (caffeic acid)^47^ (**6**) (Fig. 1). The 2D and 3D structures of each compound were sketched and constructed in the Marvin Sketch software (https://chemaxon.com/marvin), and geometry optimization was performed using the MMFF94 force field in the same software (ChemAxon, https://chemaxon.com/products/marvin).

### Py-CoMFA (3D-QSAR) modeling

A general workflow for developing a 3D-QSAR model, using the 3D-QSAR server (https://www.3d-qsar.com/), comprises uploading a dataset with the Py-MolEdit module (chemical structures and biological activity values), aligning the dataset with the Py-Align module (or optionally using a previously aligned dataset), and then build a model with the Py-CoMFA module.

The (IC_50_, μM) values of all inhibitors were converted into pIC_50_ (− LogIC_50_, M) values using the Py-MolEdit module in the 3D-QSAR server (https://www.3d-qsar.com/). The 34 selected compounds were divided randomly or by the Kennard-Stone method into two groups: 25 as training set and 9 as test set. The pIC_50_ values were used as the dependent variable (y = biological activity response), while the Py-CoMFA molecular interaction fields (MIFs) were taken as independent variables (x = descriptors). The MIFs are calculated at each grid point of a virtual cubic box by means of a predefined probe atom as steric (STE) and electrostatic (ELE) interaction fields by means of the Lennard–Jones and Coulomb potentials, respectively, and also as a combined steric and electrostatic (STE + ELE) field.

We analyzed the conformations of all inhibitors using the RDkit method applying UFF force field, max attempts of 1000, prune RMS threshold = 0.1, cluster method by RMSD considering 2.0 Å and 50 iterations, through the Py-ConfSearch platform (https://v2.3d-qsar.com/v2/pyconfsearch). We selected the most extended conformation of each inhibitor. The compound with the highest pIC_50_ (i.e., lowest IC_50_) value among all the inhibitors was selected as a template for the conformer alignment of other compounds, using Py-Align platform (https://v2.3d-qsar.com/v2/pyalign) applying RDkit method using the most extended conformation of the most active inhibitor. The Py-CoMFA steric (STE), electrostatic (ELE), and combined (STE + ELE) molecular interaction fields were calculated through the 3D-QSAR server (https://v2.3d-qsar.com/v2/pycomfa/) using the following parameters: probes atoms and charges = C*sp*^3^ (+ 1), O*sp*^3^ (− 1), and H (+ 1); grid spacing = 2.0 Å; grid extension = 5.0 Å; cutoff energy = 30 kcal/mol; minimum sigma = 2 Å; Gasteiger charge model; and a maximum number (N) of principal components (PC) = 15. The efficiency of the Py-CoMFA (3D-QSAR) model was determined by the partial least square (PLS) regression technique.

Internal validation was performed using the leave-one-out cross-validation (LOO-cv) method and applying the Y-randomization test. The cross-validation (*q*^*2*^) and quadratic linear correlation coefficient (*r*^*2*^) analysis were used to select the best model. Statistical analyses of Pearson’s correlation and F-test between models were performed using the GraphPad software (version 8.00) (GraphPad Software, Inc., USA, 6.00) – ANOVA.

To assess the applicability domain, we employed two methods: k-nearest neighbors (kNN) using Euclidean distances as metric and Leverage^53–55^.

### Designing of new compounds

Based on the information obtained from the contour maps of the best predictive Py-CoMFA model, 23 new compounds (Fig. 5) were designed by substitution of specific substituent groups (CH_3_, OH, NH_2_, OCH_3_, F and OCO_2_CH_3_) with different degrees of inductive and/or resonance effects as electron donor or acceptor in the R1-position of the phenyl ring, and in the R2-position we tested the presence of the cyclohexyl, pyrrole and indole rings. The pIC_50_ values of the newly designed compounds were predicted using the best predictive constructed Py-CoMFA model.

### Molecular docking

We used the structural 3D model of the *Leishmania* (*L*.) *amazonensis* arginase (*La*ARG) (UniProt accession number: O96394) built previously by Camargo et al.^69^ using comparative (homology) modeling and as a template the crystal structure of the *Leishmania mexicana* arginase enzyme in complex with the catalytic product (L-ornithine) (PDB ID: 4IU5, Resolution: 1.95 Å). The model is available in ModelArchive at 10.5452/ma-a8uzk. *Leishmania* (*L*.) *amazonensis* is a member of the Leishmania mexicana complex, which can cause all types of leishmaniasis infections. The molecular docking was performed using the GOLD (v. 2022.3) (Genetic Optimization for Ligand Docking) program, applying the ChemPLP function score^70^.

The FTMap family of web servers (https://ftmap.bu.edu/) was used to detect and characterize hot spot amino acid residues as potential binding sites^58^, revealing two regions for the *La*ARG protein model to be evaluated by molecular docking: the active site and an allosteric site. The *La*ARG active site region, including the detected hot spot residues and two Mn(II) cations, was centered on the Cartesian coordinates (x = 15.5865, y =  − 14.5855, z =  − 3.9095) in the middle of Mn(II)(1) and Mn(II)(2) cations. The *La*ARG allosteric site region was centered on the Cartesian coordinates (x = 12.0600, y =  − 5.7110, z =  − 16.3440) of the center of mass of the detected hot spot residues. The radius (r) of both binding sites was set to 15 Å. The ligands were subjected to 10 iterative docking runs. We performed clustering analysis to identify probable best-scored pose results. The analysis of intermolecular interactions was carried out using the Discovery Studio Visualizer program (Dassault Systèmes BIOVIA, Discovery Studio Modeling Environment, Release 2017, San Diego: Dassault Systèmes, 2016) and the figures were constructed using the Visual Molecular Dynamics (VMD) program (http://www.ks.uiuc.edu/Research/vmd/)^71^.

### Molecular dynamics simulations

The molecular dynamics simulations were performed using the GROMACS version 5.1.4 and 2019 package^72,73^, applying the CHARMM36 all-atom (AA) force field^60^ and TIP3P water model with the protein–ligand poses (complexes) from molecular docking. The atoms order was corrected by sort_mol2_bonds.pl Python script and ligand topology acquired through the CHARMM General Force Field for organic molecules (CGenFF) server (https://cgenff.umaryland.edu/initguess/).

The ionization states of the protein’s residues were adjusted to pH 7.4 using the pdb2gmx Python script. Each complex was centered inside a cubic periodic box (4.374 × 3.702 × 3.504 nm, volume = 453.95 nm3), solvated with water TIP3P type, and neutralized with Na^+^ ions. The residues Asp125, Asp129, Asp231, Asp233, His102, His127, and His142 coordinated with metal ions and had their distance restraints and constraints included in protein topology.

Systems were submitted to energy minimization steps using a convergence criterion of 1000 kJ mol^−1^. nm^−1^ followed by gradient conjugate until 100 kJ mol^−1^.nm^−1^. The minimized systems were submitted to two equilibration steps, considering 300 K of temperature (V-rescale thermostat) and 1 bar of pressure (Parrinello-Rahman barostat), realized during 1 ns and position restraint for enzyme and ligand. First, considering volume, and temperature constant (NVT ensemble), and then considering the system as isothermal-isobaric (NPT ensemble). Hydrogen atoms in the system were frozen using the LINCS algorithm, and the long-distance electrostatic interactions using the PME algorithm with a cut-off radius of 1 nm were applied to the van der Waals and Coulomb interactions. After the thermalization step, the molecular dynamics (MD) simulations were performed during 200 ns, using 2 fs integration time and 20 Å of cut-off radius to the long-distance interactions.

The *La*ARG-ligand complexes in aqueous systems were evaluated by the root mean square deviations (RMSD) and root mean square fluctuation (RMSF), the hydrogen bonding (H-bond) intermolecular interactions were computed considering the cut-off until 0.50 nm by the package of GROMACS 2019. The H-bonds frequency was calculated using the hbmap2grace package^74^, and the graphs were plotted using the XMGRACE 5.1.19 program^75^.

The binding free energy (ΔG_bind_) between the ligands and the *La*ARG model was calculated using the molecular mechanics Poisson-Boltzmann surface area (MM-PBSA) method through the module added to the GROMACS 5.1.4 program package. The contribution of residues to the binding energy was computed by MmPbSaDecomp.py Python script. Figures were constructed using the Visual Molecular Dynamics (VMD) program (http://www.ks.uiuc.edu/Research/vmd/).

### In silico predictions of pharmacokinetic and toxicity parameters (ADMET)

In silico pharmacokinetic (absorption, distribution, metabolism, and excretion, ADME) parameters related to oral bioavailability (drug-likeness) of the compounds were assessed by rule-based filters from Lipinski^76^ and Veber using ADMETlab 2.0 platform (https://admetmesh.scbdd.com/). In silico toxicity predictions were performed using the OSIRIS Property Explorer (https://www.organic-chemistry.org/prog/peo/) platform.

## Conclusions

The study utilized 3D-QSAR (Py-CoMFA) and other molecular modeling techniques, such as molecular docking and molecular dynamics simulations, to analyze how arginase inhibitors from *L.* (*L.*) *amazonensis* interact and their affinity. The pIC_50_ values calculated with the best Py-CoMFA model were in excellent correlation with the experimental values. The molecular docking study identified the favorable hydrophobicity contribution of phenyl and cyclohexyl groups as substituents in the cinnamide compounds. These groups can act as pharmacophoric groups in designing new inhibitors. The study showed that derivatives interacted more effectively with the allosteric site of the protein rather than the active site. The results identified two compounds which can guide the development of more effective arginase inhibitors for the treatment against leishmaniasis.

### Supplementary Information

Supplementary Information.

## Data Availability

The authors confirm all data generated and analyzed during this study are available in the article and in the supplementary information.
