# Supplementary material for: Py-CoMFA, docking, and molecular dynamics simulations of Leishmania (L.) amazonensis arginase inhibitors
Source: Sci Rep. 2024 May 21;14:11575. doi: 10.1038/s41598-024-62520-2 (PMC11109165; doi:10.1038/s41598-024-62520-2)
Supplement: Supplementary file 1 — Supplementary Information. [file 41598_2024_62520_MOESM1_ESM.docx]

**Py-CoMFA, Docking, and Molecular Dynamics Simulations of *Leishmania (L.) amazonensis* Arginase Inhibitors**

Priscila Goes Camargo ^a^, Carine Ribeiro dos Santos ^b^, Magaly Girão Albuquerque ^b^,
Carlos Rangel Rodrigues ^a*^, Camilo Henrique da Silva Lima ^b*^

*^a^ Faculdade de Farmácia, Departamento de Fármacos e Medicamentos, Universidade Federal do Rio de Janeiro, Rio de Janeiro, RJ, Brazil.*

*^b^ Laboratório de Modelagem Molecular (LabMMol), Instituto de Química, Universidade Federal do Rio de Janeiro, Rio de Janeiro, RJ, Brazil.*

**ORCID Authors:**

Priscila G. Camargo 0000-0003-4483-2119

Carine R. dos Santos 0000-0002-6436-9101

Magaly G. Albuquerque 0000-0003-1558-0928

Carlos R. Rodrigues 0000-0001-8453-7654

Camilo Henrique da S. Lima 0000-0001-8453-7654

*** Corresponding authors**

E-mail address: camilolima@iq.ufrj.br (C. H. S. L), rangelfarmacia@gmail.com (C. R. R.)

**SUPPORTING INFORMATION**

**Table S1.** Statistical parameters of the Py-CoMFA (3D-QSAR) models built using electrostatic (ELE) and combination of steric and electrostatic fields (STE+ELE) and 2 Å grid spacing, including Y-randomization (Y-r) test, according to the training and test sets splitting method (Kennard-Stone) and the probe atoms and charges (C*sp*^3^, +1; O*sp*^3^, −1; and H, +1).

| **Models** | **ELE** | **STE+ELE** | **ELE** | **STE+ELE** | **ELE** | **STE+ELE** |
| --- | --- | --- | --- | --- | --- | --- |
| **Probe atom** | C*sp*^3^ | C*sp*^3^ | O*sp*^3^ | O*sp*^3^ | H | H |
| **Charge** | +1 | +1 | −1 | −1 | +1 | +1 |
| **r^2^** | 0.098 | 0.159 | 0.098 | 0.163 | 0.098 | 0.162 |
| **SDEC** | 0.645 | 0.623 | 0.645 | 0.622 | 0.645 | 0.622 |
| **q^2^** | -0.179 | -0.118 | -0.179 | -0.122 | -0.179 | -0.125 |
| **SDEP** | 0.738 | 0.718 | 0.738 | 0.720 | 0.738 | 0.721 |
| **r^2^ (Y-r)** | 0.116 | 0.417 | 0.116 | 0.417 | 0.116 | 0.417 |
| **SDEC (Y-r)** | 0.639 | 0.518 | 0.639 | 0.519 | 0.639 | 0.519 |
| **q^2^ (Y-r)** | -0.032 | 0.285 | -0.032 | 0.283 | -0.032 | 0.283 |
| **SDEP (Y-r)** | 0.690 | 0.574 | 0.690 | 0.575 | 0.690 | 0.575 |
| r^2^ = coefficient of determination; SDEC = standard deviation error in calculation; q^2^ = r^2^ from leave-one-out (LOO) cross-validation; SDEP = standard deviation error in prediction. | | | | | | |


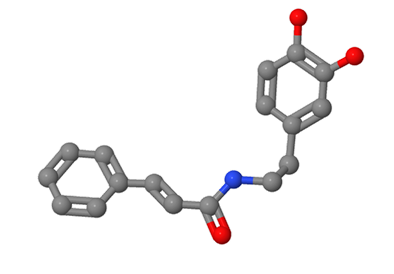

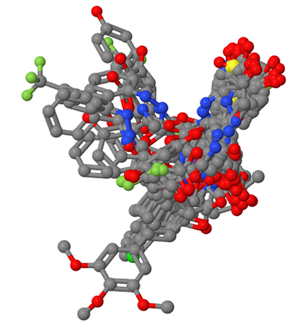


**B**

**A**

**Figure S1. (A)** The longest conformation, **4h**, was used as an alignment template. **(B)** Superimposed conformation structures of all inhibitors under study resulted in molecular docking results. Compounds are shown in the ball-and-stick model, and the atoms are color-coded as follows: carbon (grey), oxygen (red), nitrogen (blue), fluorine (green), and sulfur (yellow). Hydrogen atoms were omitted for clarity.

**C**

**B**

**A**

**Figure S2.** Experimental *versus* predicted pIC_50_ values for the training and test set compounds according to the Py-CoMFA (3D-QSAR) models built using steric field, 2 Å grid spacing, and the Kennard-Stone training and test sets splitting method by probe atom and charges C*sp*^3^ (+1): **(A)** STE model field (y = 0.3595x + 3.166; R² = 0.360); **(B)** ELE model field (y = 0.3861x + 3.035; R² = 0.386); and **(C)** STE+ELE model field (y = 0.9635x + 0.1802; R² = 0.963).

**Table S2.** Experimental pIC_50_ (pIC_50Exp_), predicted pIC_50_ (pIC_50Pred_), and residual (Res = pIC_50Exp_ − pIC_50Pred_) values for the training and test sets compounds based on the Py-CoMFA models built using steric field, 2 Å grid spacing, and the Kennard-Stone training and test sets splitting method, as probe atoms C*sp*^3^ (charge = +1).

| **Parameters** | **STE** | **ELE** | **STE+ELE** |
| --- | --- | --- | --- |
| **Probe atom** | C*sp*^3^ | C*sp*^3^ | C*sp*^3^ |
| **Charge** | +1 | +1 | +1 |
| **r^2^** | 0.359 | 0.386 | 0.964 |
| **SDEC** | 0.554 | 0.542 | 0.132 |
| **q^2^** | -0.280 | 0.137 | 0.340 |
| **SDEP** | 0.783 | 0.643 | 0.562 |
| **F-test** | 12.91 | 14.46 | 609 |
| ***r*** | 0.599 | 0.621 | 0.981 |
| **r^2^ (Y-r)** | 0.436 | 0.443 | 0.880 |
| **SDEC (Y-r)** | 0.520 | 0.516 | 0.240 |
| **q^2^ (Y-r)** | 0.204 | 0.047 | 0.152 |
| **SDEP (Y-r)** | 0.617 | 0.675 | 0.637 |

Models presented a P value <0.0001. r^2^ = coefficient of determination; SDEC = standard deviation error in calculation; q^2^ = r^2^ from leave-one-out (LOO) cross-validation; SDEP = standard deviation error in prediction; *r* = Pearson correlation coefficient.

**Table S3.** Experimental pIC_50_ (pIC_50Exp_), predicted pIC_50_ (pIC_50Pred_), and residual (Res = pIC_50Exp_ − pIC_50Pred_) values for the training and test sets compounds (aligned by compounds conformation from docking results) based on the Py-CoMFA STE+ELE model built using steric field, 2 Å grid spacing, and the Kennard-Stone training and test sets splitting method, as probe atoms C*sp*^3^ (charge = +1).

| **Classes** |  |  | **C*sp*^3^** (+1) | |
| --- | --- | --- | --- | --- |
|  |  | **pIC_50Exp_** | **pIC_50Pred_** | **Res** |
| **Chromones** | **1a** | 5.854 | 5.575 | 0.279 |
|  | ***1b** | 5.046 | 5.206 | -0.160 |
|  | **1c** | 5.310 | 5.585 | -0.275 |
|  | **1d** | 5.260 | 5.298 | -0.038 |
| **Pyrimidines** | **2a** | 4.921 | 4.785 | 0.136 |
|  | **2b** | 4.000 | 4.104 | -0.104 |
|  | **2c** | 4.000 | 4.048 | -0.048 |
|  | **2d** | 4.000 | 4.094 | -0.094 |
|  | ***2e** | 4.000 | 4.733 | -0.733 |
|  | ***2f** | 4.328 | 4.659 | -0.331 |
| **Phenylhydrazines** | **3a** | 4.921 | 4.572 | 0.349 |
|  | **3b** | 4.921 | 4.848 | 0.073 |
|  | **3c** | 4.420 | 4.494 | -0.074 |
|  | **3d** | 4.000 | 3.962 | 0.038 |
|  | **3e** | 4.000 | 3.970 | 0.030 |
|  | **3f** | 4.432 | 4.531 | -0.099 |
| **Cinnamides** | **4a** | 5.161 | 5.326 | -0.165 |
|  | **4b** | 5.252 | 5.153 | 0.099 |
|  | ***4c** | 5.745 | 5.471 | 0.273 |
|  | ***4d** | 5.187 | 5.372 | -0.185 |
|  | **4e** | 5.602 | 5.574 | 0.028 |
|  | ***4f** | 4.745 | 4.844 | -0.099 |
|  | ***4g** | 5.046 | 5.593 | -0.548 |
|  | **4h** | 5.886 | 5.825 | 0.062 |
|  | ***4i** | 5.398 | 5.771 | -0.373 |
|  | **4j** | 5.260 | 5.209 | 0.050 |
|  | **4k** | 5.620 | 5.741 | -0.121 |
| **Cinnamic esters** | **5a** | 5.553 | 5.566 | -0.013 |
|  | **5b** | 5.523 | 5.619 | -0.096 |
|  | **5c** | 5.721 | 5.694 | 0.027 |
|  | **5d** | 3.699 | 3.665 | 0.034 |
|  | **5e** | 4.444 | 4.588 | -0.144 |
|  | ***5f** | 5.678 | 5.693 | -0.015 |
| **Cinnamic acid** | **6** | 5.824 | 5.758 | 0.066 |

***Supplementary data for allosteric site mapping by FTMap program***

Residue interactions by hydrogen bonding and hydrophobic interactions greater than 5% were considered to analyze clusters generated by the mapping (Figure S3). The residues that showed the highest percentage of hydrogen bonding and hydrophobic interaction were Trp40, Phe94, Gly100, Asp129, Ser138, His142, and Leu281 (Figure S3). Except for Gly100, Asp129, Ser138, His142, and Leu281 residues that belong to the active site already evaluated, the other residues (Lys1, Lys2, Met3, Ser4, Leu96, Leu269, Val270, and Met302) until 10 Å from Trp40, Phe94 (Figure S3) were considered for analysis within the protocol adopted for docking the allosteric site.


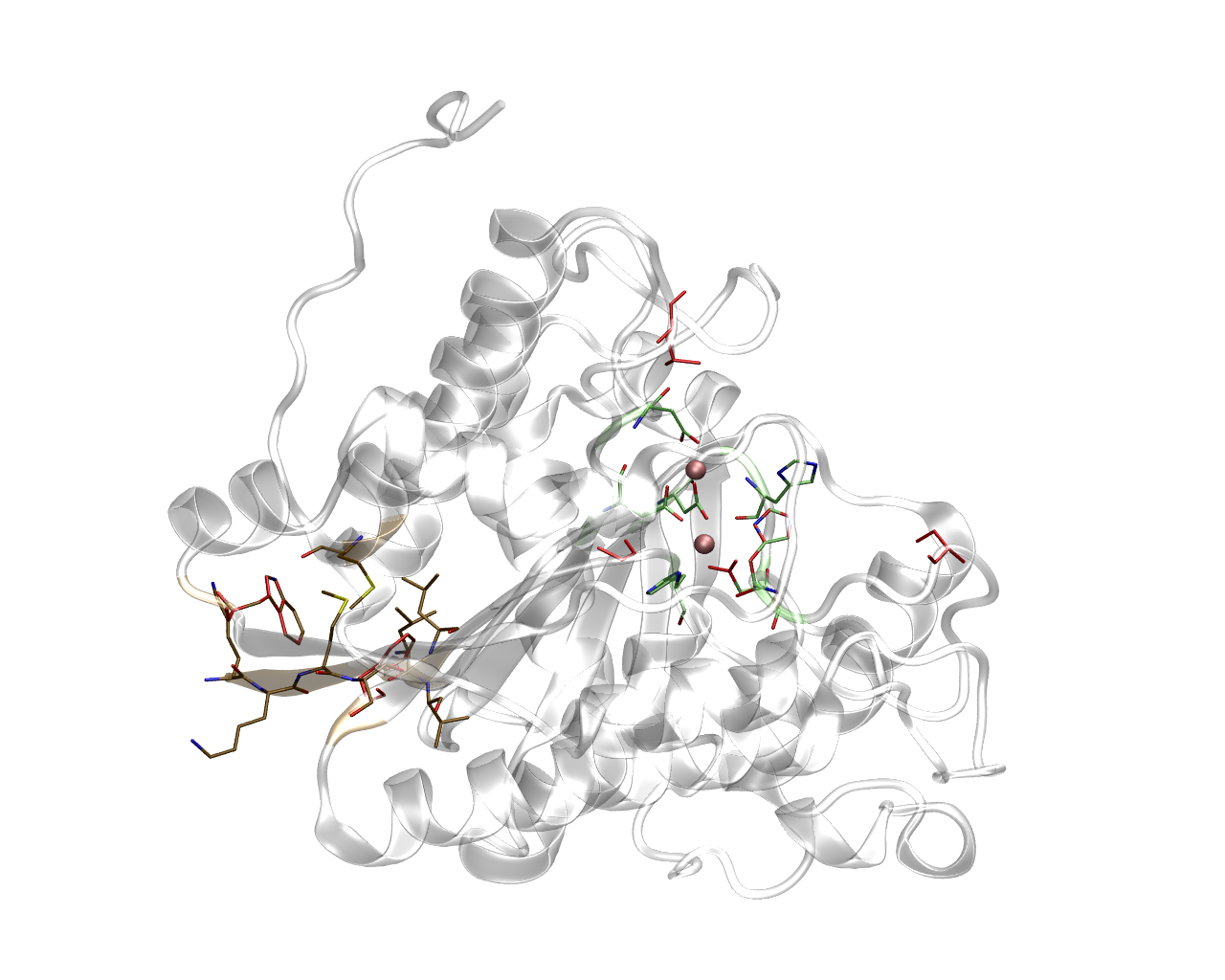


**Figure S3.** FTMap possible interaction sites to LaARG. The green color represents the active site, and the ochre color represents the allosteric site; residues more significant than 5% of interactions are highlighted in red (shown for a monomer).
